# “Therapeutic potential of *Acalypha indica* L. leaf fractions against foodborne pathogens: an in vitro and in silico study”

**DOI:** 10.1038/s41598-025-32216-2

**Published:** 2025-12-19

**Authors:** Uma Venkatesan, Rajiniraja Muniyan

**Affiliations:** School of Bio - Sciences and Technology, Vellore Institute of Technology, Vellore, 632014 India

**Keywords:** Antimicrobial activity, Response surface methodology, GC-MS, Molecular docking, Molecular dynamics, Biochemistry, Biotechnology, Chemical biology, Chemistry, Computational biology and bioinformatics, Drug discovery, Microbiology

## Abstract

This study investigates the inhibition effects of *Acalypha indica* L. leaf extract obtained using various solvents, viz. petroleum ether, chloroform and ethanol. Among the extracts, the ethanolic extract showed the strongest antioxidant activity, with 84.36% scavenging potential in 2,2-diphenyl-1-picrylhydrazyl (DPPH) and 69% in the Ferric ion Reducing Antioxidant Power (FRAP) assay. Similarly, in antimicrobial activity, the ethanolic extract showed the highest inhibition zone of 24.0 mm against *S. aureus* and 29.3 mm against *E. coli.* Response Surface Methodology (RSM) utilize a Box-Behnken design (BBD) analysed with optimize conditions for enhancing antioxidant activity. Gas Chromatography-Mass Spectrometry (GC-MS) identified eleven major phytocompounds, which were further evaluated through molecular docking and ADMET studies against *S. aureus’s* DNA gyrase B and *E. coli’s* Dihydrofolate reductase. Docking result shows highest binding affinity towards two compounds such as 2(5 H)-Furanone,3-chloro-5-((dimethylamino)methyl)-4,5-dimethyl- and N-(2,2-Dichloro-1-hydroxyethyl)-2,2-dimethylpropanamide with docking scores of -5.21 and − 8.38 kcal/mol for *S. aureus* and − 4.39 and − 8.75 kcal/mol for *E. coli*. These two hits were selected for molecular dynamic simulation studies to evaluate protein-ligand complex stability. Overall, the ethanolic extract exhibited strong antioxidant and antimicrobial properties, suggesting as potential candidate for application in food packaging.

**Supplementary Information:**

The online version contains supplementary material available at 10.1038/s41598-025-32216-2.

## Introduction

Food is the primary source of energy for humans but is highly vulnerable to microbial contamination, leading to food spoilage and health risks. Foodborne illnesses represent a considerable threat to global public health, affecting approximately 7.7% of the global population each year, with a mortality rate of about 7.5%^1^. The increasing development of antibiotic resistance further intensifies the need for alternative antimicrobial strategies. Although synthetic preservatives are highly effective in controlling microbial growth, they present drawbacks such as chemical residues in food, the emergence of resistant microbial strains, and adverse health effects, which has limited consumer acceptance and prompted research into natural alternatives^2^. Green synthesis has emerged as an efficient and cost-effective approach that minimizes the need for additional stabilizing components while remaining ecologically friendly^3,4^. The World Health Organization (WHO) indicates that more than 80% of the global population based on traditional and folk remedies, primarily based on herbal products^5^.

Medicinal plants have long been used to treat health ailments, enhance flavor, preserve food, and combat infections, and their derivatives-including extracts, essential oils, powders, and bioactive compounds are increasingly recognized for their efficiency in food bio-preservation by maintaining sensory and microbiological properties while extending shelf life^6^. The WHO reports that there are approximately 20,000 medicinal plants globally, with India contributing about 15–20% of this total. Moreover, around 2,000 therapeutic formulations in India are derived from plants. Even in technologically advanced countries such as the United States, about 25–40% of the drugs listed in pharmacopoeias are of plant origin^7^. Plant extracts represent promising candidates for the development of novel antibacterial therapeutics due to their diverse bioactive compounds, which have demonstrated significant antibacterial efficacy. Plant-derived components have also gained increasing attention as natural and environmentally friendly alternatives for controlling foodborne infections^8,9^. Among these, *Acalypha indica* has attracted interest for its well-documented health benefits and antibacterial properties. For instance, studies on *Alhagi maurorum* leaves revealed significant scavenging capacity attributed to phenolic substances, highlighting the potential of green synthesis processes that are not only cost-effective and accessible but also compatible with biological applications^10^.

*Acalypha indica* L. belongs to the family Euphorbiaceae and is commonly referred to as *kuppaimeni* among the native populace of Tamil Nadu. It is a weed species widely distributed across the plains of India. Traditionally, *A. indica* has been used to treat several ailments, including respiratory infections, breathing problems, and arthritis, and it exhibits antivenom, anti-inflammatory, antimicrobial and antioxidant properties^11^. However, most previous studies have focused on crude extracts, with limited efforts toward isolating or evaluate partially purified fractions that may contain more potent bioactive compounds. Moreover, the potential of these fractions in food preservation applications and their molecular interactions with essential antimicrobial target proteins remain largely unexplored.

The role of binding pockets of *Staphylococcus aureus* DNA Gyrase B (GyrB) and *Escherichia coli* dihydrofolate reductase (DHFR) were utilized as documented in a prior investigation. DNA gyrase represents potential target for antibacterial drugs. Among the two subunits of DNA gyrase, GyrA facilitates DNA double-strand cleavage, while the GyrB subunit is liable for ATPase action. The mechanisms of antibiotics that inhibit DNA gyrase involve targeting distinct domains of its two subunits. DNA gyrase B is a subunit of the bacterial enzyme that creates a tetramer with GyrA subunits. It is crucial for DNA supercoiling via ATP binding and hydrolysis, enabling the transit of the strands of DNA while replication and transcription. It encompasses an ATP binding pocket and is integral to the enzyme’s catalytic function. Another protein, DHFR is a metabolic enzyme in the folic acid system that catalyzes the conversion of dihydrofolate to tetrahydrofolate, essential for cell proliferation through thymidine production. It is a pivotal gene product, particularly in the eradication of antibiotic-resistant bacteria^12^.

**Fig. 1:**
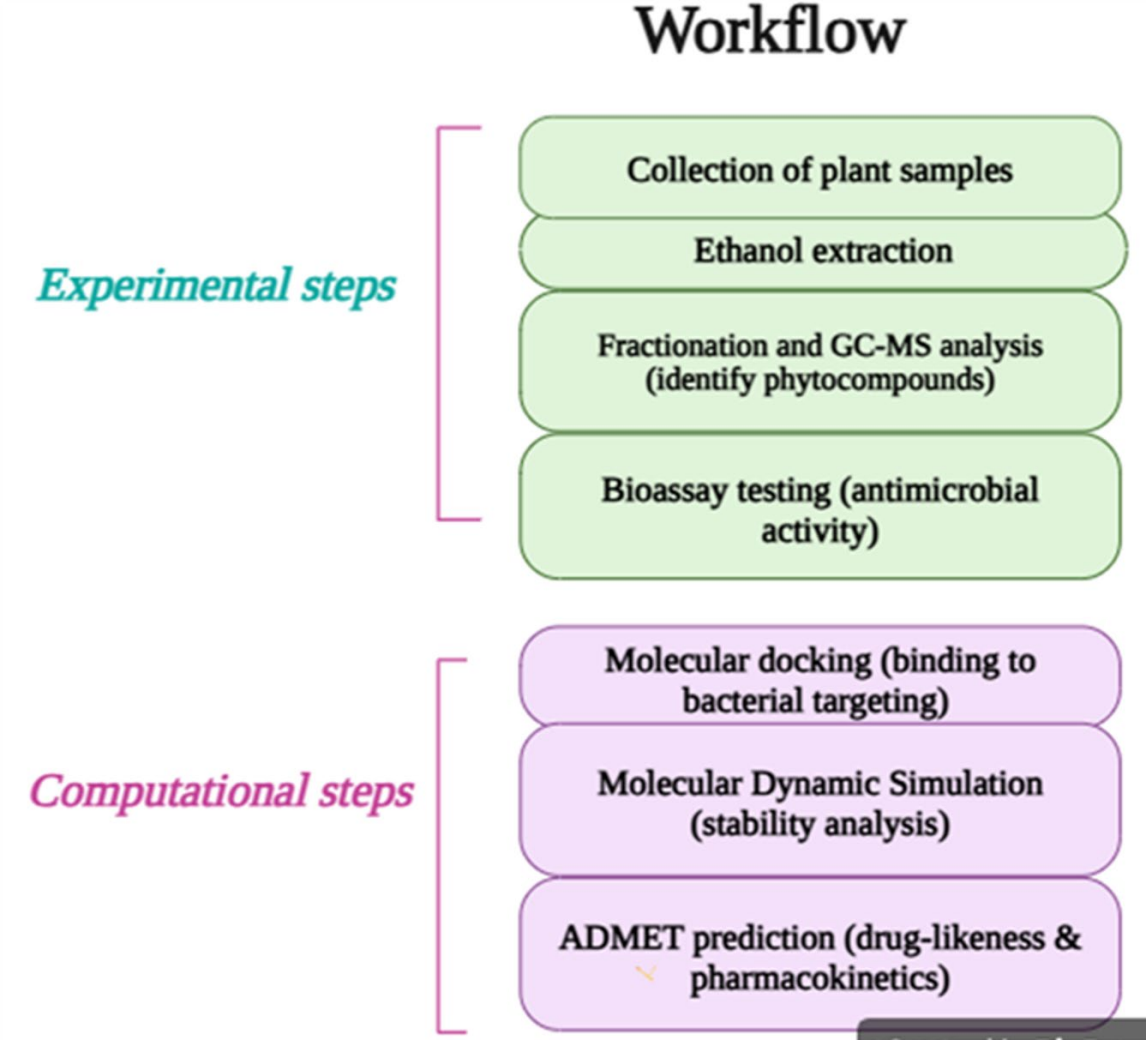
An overview of the study workflow illustrating the extraction, fractionation, GC-MS analysis, bioassays and computational analyses (molecular docking, molecular dynamics and ADMET analyses) of *Acalypha indica* leaf extract.

The overall workflow of the research summarizes the experimental and computational aspects in Fig. 1. This process involves the extraction and fractionation of *Acalypha indica* leaf extract, chemical profiling via GC-MS analysis, biological analysis through antioxidant and antibacterial bioassays, and computational assessments that include molecular docking, molecular dynamics (MD) simulations and ADMET projections.

Therefore, the current study investigates a partially purified ethanol extract of *A. indica* leaf extract using a combined in vitro and in silico approach against foodborne pathogens. The study integrates antioxidant and antibacterial analyses with molecular docking, ADMET evaluation, and molecular dynamics simulations to address existing research gaps. These combined methodologies provide novel insights into the mode of action of *A. indica* compounds and their possibility application as natural preservatives in food packaging systems.

## Experimental part

### Chemicals and reagents

Both the chemicals and reagents were used in the experiments, which consisted of analytical grade and were acquired from Merck, Himedia and Sigma Aldrich.

### Collection of plant sample and soxhlet’s extraction

The field of research in the present study was conducted in compliance with the relevant institutional norms and regulations. No endangered or protected species were involved, and the work adhered to both institutional and national guidelines. Fresh leaves of *A. indica* were collected from the campus of VIT University, Vellore, Tamil Nadu, India. The plant material was authenticated, and herbarium specimens were prepared and deposited by Dr. C. Rajasekaran, Department of Plant Biotechnology, VIT University (voucher specimen no. *Acalypha indica* - VITCS02-1).

The collected plant leaves were washed with running water to eradicate any impurities, then cleaned with distilled water and dried with air in a shade for a period of two weeks. The dried-out material is made into powder using a mixer and kept in an open area for drying at ambient temperature. The powdered substances are placed in an airtight glass container prior to extraction with an organic solvent. The petroleum ether, chloroform and ethanol were chosen as extraction solvents because of their diverse polarity, which facilitates the extraction of a wide range of phytocomponents from non-polar to moderately polar components. Petroleum ether (non-polar) facilitated the separation of fatty acids, lipids and terpenoids, chloroform (moderately polar) retrieved alkaloids and specific phenolic compounds, and ethanol relatively safe and effective solvent with a strong ability the extraction of polar constituents including phenolic acids, tannins, and flavonoids, glycosides. Methanol was excluded due to its higher toxicity, while water was not chosen because it predominantly removes highly polar components which may lead to reduced recovery of bioactive components. Dried powder (25 g) was extracted using solvents (300 mL) of increasing polarity using a Soxhlet apparatus. The boiling point of solvents and corresponding extraction temperatures were as follows: petroleum ether (40–60 °C; ~50 °C), chloroform (61–62 °C; ~60 °C), and ethanol (78 °C; ~78 °C) and each extraction was subjected into 6 to 8 h with a Soxhlet extractor. The different extracts were filtered through Whatman No. 1 filter paper and further dried with a rotary evaporator, and the yield was measured^13–15^.

### Evaluation of antioxidant assay

Chemical-based studies can be categorized into various techniques for evaluating antioxidant activity including methods that determine scavenging activity on stable free radicals (2, 2-diphenyl-1-picrylhydrazyl (DPPH)) and those that assess the minimize of metal ions (Ferric ion Reducing Antioxidant Power (FRAP). The use of a distinct antioxidant activity assessment technique is ineffective for evaluating the oxidative stress inhibitory capacity of the samples.

#### DPPH radical scavenging assay

To assess the extract’s capacity to scavenge free radicals and the DPPH study was carried out earlier methods with a few modifications. Briefly, 0.1 mL extract (1 mg/mL) at varying concentrations (50, 100, 150, 200 and 250 µg/mL) along with the ascorbic acid as a standard and added with a 0.1 mM methanolic solution of DPPH dissolved in a constant volume of DPPH (3.9 mL) solution to evaluate the percentage radical scavenging activity of plant extracts. To determine the plant extracts’ potential to scavenge free radicals and the absorbance was noted at 517 nm utilizing a spectrophotometer^16^.

$$\:\mathrm{\%}\:\mathrm{o}\mathrm{f}\:\mathrm{D}\mathrm{P}\mathrm{P}\mathrm{H}\:\mathrm{s}\mathrm{c}\mathrm{a}\mathrm{v}\mathrm{e}\mathrm{n}\mathrm{g}\mathrm{i}\mathrm{n}\mathrm{g}\:\mathrm{a}\mathrm{c}\mathrm{t}\mathrm{i}\mathrm{v}\mathrm{i}\mathrm{t}\mathrm{y}\:=\frac{absorbance\:of\:control-absorbance\:of\:sample}{absorbance\:of\:control}\:\times\:100$$

Absorbance of sample = DPPH + methanol + plant extract.

Absorbance of the control = DPPH + methanol.

### FRAP assay

The ferric reducing or antioxidant capacity was evaluated as previously reported with slight modifications. 100 µL of the extract of different concentrations (50, 100, 150, 200 and 250 µg/mL) were combined with 2.5 mL of 200 mmol per Liter phosphate buffer solution (pH level 6.6) and 2.5 mL of 1% potassium ferricyanide, followed by incubation at 50 °C for 20 min. Subsequently, a volume of 2.5 mL of 10% trichloroacetic acid was then added and the test tubes were centrifuged at 10,000 rpm for a duration of ten minutes. Subsequently, 5 mL of the first layer was combined with 5.0 mL of distilled water and 1 mL of 0.1% ferric chloride, after which the absorbance was measured at 700 nm. The ascorbic acid was utilized as a positive control^17^. Afterwards, the FRAP value can be calculated to the following formula.

$$\:\mathrm{\%}\:\mathrm{F}\mathrm{R}\mathrm{A}\mathrm{P}\:\mathrm{a}\mathrm{s}\mathrm{s}\mathrm{a}\mathrm{y}\:=\frac{absorbance\:of\:sample-absorbance\:of\:control}{absorbance\:of\:sample}\:\times\:100$$

Absorbance of sample = FRAP reagent + plant extract.

Absorbance of the control = FRAP reagent without plant extract.

### In vitro antimicrobial assay

The effectiveness of the antibacterial of identified plants was explored into the agar well diffusion method. Two bacterial strains were used as test organisms includes *Staphylococcus aureus* (MTCC 1144) and *Escherichia coli* (MTCC 452).

#### Agar well diffusion technique

The antimicrobial effects of *A. indica* different solvent extracts were evaluated by applying the agar well diffusion technique as discussed but slightly modified. Utilizing a sterile cork borer, four wells were developed in the agar plate and thereafter filled with 30 µL of the different concentration (10, 15, 20, and 25 mg/mL) and positive control (tetracycline) (1 mg/mL). Then, antimicrobial plates were incubated at 37 °C for 24 h and experiments were done in triplicates (*n* = 3). The measurement of the diameter, the zone of inhibited growth, was calculated, and the average values were estimated^18^.

### Antibiofilm assay

Selected effective ethanol extract estimates for their antibiofilm activity against *S. aureus* and *E. coli* were tested using a microtiter plate assay to determine biofilm elimination with minor modifications. A suspension of bacteria (1 × 10⁶ CFU/mL) was maintained in nutrient broth and 100 µL was given to each well of a 96-well flat-bottom microplate. A negative control (DMSO) and a positive control (tetracycline), both containing only microorganisms were embedded. The wells were filled with equal volumes of ethanol extract at different concentrations (50, 100, 150, 200 and 250 µg/mL). The plates were then incubated at 37℃ for 24 h to promote the growth of biofilm and experiments were done in triplicates (*n* = 3). After incubation, a crystal violet (CV) assay was used to evaluate the biofilm inhibition. Then, the wells are washed thrice with phosphate buffer saline (PBS) and dried at ambient temperature. Each well was stained with 0.1% of crystal violet and allow to stand for 15 min to fix adherent cells. Further, the wells are washed with PBS thrice to remove any unbound cells. Finally, 30% glacial acetic acid were added and examined under ultraviolet (UV) light spectrophotometer at 590nm^19^. Afterwards, the inhibition value can be calculated as following formula.

$$\:\mathrm{\%}\:\mathrm{B}\mathrm{i}\mathrm{o}\mathrm{f}\mathrm{i}\mathrm{l}\mathrm{m}\:\mathrm{i}\mathrm{n}\mathrm{h}\mathrm{i}\mathrm{b}\mathrm{i}\mathrm{t}\mathrm{i}\mathrm{o}\mathrm{n}\:\mathrm{a}\mathrm{s}\mathrm{s}\mathrm{a}\mathrm{y}\:=\frac{absorbance\:of\:control-absorbance\:of\:sample}{absorbance\:of\:control}\:\times\:100$$

Absorbance of sample = microorganisms with plant extract.

Absorbance of control = only microorganism without plant extract.

### Optimization study using response surface methodology (RSM)

 The current research implemented a three-factor and three-level Box-Behnken design (BBD) to estimate the optimal conditions for maximizing the antioxidant activity of *A. indica* ethanol extract in both DPPH and FRAP assays with a few modifications to the standard protocol. BBD was selected due to its effectiveness in possibly incorporating three variables during the experimental domain, assisting a reduced number of tests for a less costly and more effective technique. RSM was employed to ascertain the optimum values of extraction time (X1, 30–60 min), sample concentration (X2, 50–250 µg/mL), and ethanol concentration (X3, 50–90%)^20,21^. Experimental information on DPPH and FRAP assays was adapted to a second-order polynomial model, and comprehensive model equations, coded variables, and statistical parameters (Tables S2-S5) are included in the Supplementary Information.

### Phytochemicals analysis of ethanol extract

#### Determination of total phenolic content (TPC)

The Folin-Ciocalteu technique was used to estimate the ethanol extract for the TPC with slight modifications. 100 µL of extract (1 mg/mL concentration) and 500 µL of 10% Folin-Ciocalteu reagent were mixed and the reaction was left at room temperature for five minutes. Then, 400 µl of 7.5% sodium carbonate solution was added and the mixture was allowed to stand at room temperature for 30 min in the dark. At 765 nm, the absorbance was determined with a UV-Vis spectrophotometer. Using gallic acid as a standard, the calibration curve evolved that indicated TPC in milligrams of gallic acid equivalents per gram of extract (mg GAE/g extract)^22^.

#### Determination of total flavonoid content (TFC)

The aluminium chloride method was evaluating the ethanol extract for the TFC with a few modifications. With 4 mL of distilled water and 200 µL of the extract (1 mg/mL concentration) mixed, add 0.3 mL of 5% sodium nitrite, and the mixture was left to react for five minutes. After that, 0.3 mL of 10% aluminium chloride was added, and 2 mL of 1 M sodium hydroxide was added 6 min later. Distilled water was used to dilute the reaction mixture, and it was thoroughly stirred. At 510 nm, the absorbance was measured with a UV-Vis spectrophotometer. The total flavonoid concentration was expressed in milligrams of quercetin equivalents per gram of extract (mg QE/g extract) and a calibration curve was developed using quercetin as the standard^23^.

### Chromatographic methods

#### Thin layer chromatography (TLC)

TLC was employed to separate the constituents of the crude ethanolic extract of *A. indica* (L.). Multiple solvents with varying polarities were employed, specifically hexane: ethyl acetate (4:1), benzene: ethyl acetate (4:1), toluene: ethyl acetate (4:1), ethyl acetate: hexane: formic acid (2:2:1), and petroleum ether: chloroform (4:1). A toluene: ethyl acetate (4:1) ratio has been employed to determine the optimal separation for chromatography, in contrast to the examination of all solvents. Implement the ethanol extract at a concentration of 1 mg/ml to the TLC plates and allow it to dry immediately. The plates were further examined under ultraviolet (UV) light at 366 nm and the R_f_ values have been calculated to ascertain the active metabolites using the following formula^24^.

R_f_ value = $$\:Distance\:travelled\:by\:solute/Distance\:travelled\:by\:solvent$$.

### Column chromatography

Column chromatography was performed on the ethanolic extract to separate the bioactive components. A glass column (40 mm × 600 mm) was filled with silica gel (60–120 mesh size) utilizing toluene as the wetting solvent. The column was first cleaned with toluene and dried prior to packing. A silica slurry was carefully poured to prevent air bubbles and ensure consistent packing, with glass wool inserted at the base. The ethanolic extract was added onto the column, and elution was performed with a gradient of toluene and ethyl acetate at a flow rate of 1 mL/min. The acquired fractions were determined by thin-layer chromatography (TLC) and antibacterial assay^25,26^.

#### Screening of antimicrobial activity of bioactive fractions

The obtained fractions were further investigated using TLC to ascertain the presence of phytocomponents. The formula used was based on the R_f_ value of each individual spot. Subsequently, the identical R_f_ values were aggregated, and dry weights were measured as well. To further evaluate the most active fractions, an antimicrobial assay utilizing the agar well diffusion method was conducted, adhering to the prior protocol with minimal adjustments^27^. Consequently, this bioactive fraction was employed for GC-MS analysis.

### GC-MS analysis of partially purified ethanol extract

GC-MS analysis of partially purified ethanol extract was estimated utilizing an Agilent 7890B gas chromatograph in conjunction with a 5977 A mass selective detector equipped with a DB-5MS capillary column (30 m × 0.25 mm × 0.25 μm). Helium provided as the carrier gas at a sustained flow rate of 1.0 mL min⁻¹. The injector temperature was established at 260 °C in split mode (split ratio 10:1) with an injection volume of 1 µL. The oven temperature was set to increase from 60 °C (maintained for 1 min) to 300 °C at a rate of 10 °C per minute and retained for 6 min. The temperatures of the ion source and transfer line were sustained at 200 °C. The mass spectrometer functioned in electron ionization (EI) mode at 70 eV, scanning the m/z range of 40–600 with a solvent delay of 1 minute^7^.

### Combination of molecular Docking and dynamic simulation studies

Molecular docking and molecular dynamics simulations were conducted to clarify a possible mechanism underneath the antimicrobial activities of the compounds of the *A. indica* bioactive fraction.

#### Molecular Docking studies

Based on a literature study, molecular docking studies were conducted on the GC-MS partially purified compounds from the ethanol extract with antimicrobial target proteins. The structures of our target proteins, DNA Gyrase B (GyrB) from *S. aureus* (PDB ID: 3U2D) and dihydrofolate reductase (DHFR) for *E. coli* (PDB ID: 2ANQ)^12^, were retrieved from the Protein Data Bank (PDB) (Source: www.rcsb.org/pdb/). The ligand structures were downloaded in SDF format from https://pubchem.ncbi.nlm.nih.gov and converted to PDB format using the Open Babel 3.3.1 tool^28^. The protein’s structure was generated utilizing AutoDock 4.2^29^. All heteroatoms and crystallographic water molecules were eliminated during the preparation process. Polar hydrogen atoms were included, and Kollman charges were allocated to the structure. The structures were acquired from the PubChem database for reference 1 (CID 9543473) and reference 2 (CID 54759160) and were analyzed for docking using AutoDock 4.2, which was subsequently changed to the pdbqt format. The reduced ligand library was subsequently utilized for virtual screening in AutoDock Vina 1.1.2^30^. The docking parameters for Auto Dock Vina 1.1.2 have been determined to assure reproducibility. For Reference 1, the grid box was centred at x = 29.540, y = 43.195, and z = 15.094 Å with dimensions of 72 × 118 × 126 points and a grid interval of 0.375 Å, fully encompassing the active site region. For Reference 2, the grid box was centred at x = 3.859, y = 3.165, and z = 21.379 Å with dimensions of 44 × 62 × 50 points and a grid interval of 0.375 Å. The docking parameters were set to exhaustiveness = 8, Number of modes = 10, and energy range = 4, while all other parameters were maintained at their default values. The interaction studies (2D/3D binding modes, hydrogen bond interactions, and hydrophobic contacts) were visualized with Discovery Studio Visualizer (DSV) (https://www.3ds.com/products/biovia/discovery-studio).

#### Docking validation

The reliability of the docking protocol was assessed through a redocking analysis of the co-crystallized ligand. The ligand has been removed from the active site and subsequently re-docked using the same docking parameters applied to the test compounds. The redocked pose successfully reproduced the experimental binding conformation within an RMSD limit of 2.0 Å, thereby validating the docking protocol^28^.

#### Pharmacokinetic properties, drug-likeness assessment and toxicity prediction

High GI absorption, blood-brain barrier (BBB) permeability, Pan-assay interference compounds (PAINs), and H-bond acceptors and donors were predicted using the SwissADME online server^31^. Furthermore, toxicity studies, particularly hepatotoxicity, carcinogenicity, immunotoxicity, cytotoxicity, and mutagenicity, were estimated in the Protox 3.0 web server^32^.

#### Molecular dynamic simulation (MDS)

We performed a further investigation of the stability of the selected hits and reference compounds utilizing GROMACS version 2023.1. The protein topology was created using pdb2gmx with the CHARMM27 force field, and the system was solubilized in a triclinic box filled with TIP3P water molecules. Ligand topologies were acquired from the SwissParam server^33^. The protein-ligand complexes were neutralized with sodium ions, thereafter, subjected to solvation, energy minimization (50,000 steps), and equilibration. The equilibration was done under NVT (constant particle number, volume, and temperature) and NPT (constant particle number, pressure, and temperature) sets for 100 ps each at 300 K and 1 bar, utilizing the V-rescale thermostat and Parrinello-Rahman barostat, with position restraints imposed on the large atoms. Productive molecular dynamics simulations were executed for 100 nanoseconds with a 2-femtosecond time step, utilizing regular boundary conditions in all dimensions. The Linear Constraint Solver (LINCS) approach constrained bond lengths, whereas long-range electrostatic interactions were examined using the Particle Mesh Ewald (PME) method with a cutoff of 1.0 nm for both Coulombic and van der Waals interactions. Post-simulation analyses, including Root mean square deviation (RMSD), Root mean square fluctuations (RMSF), Radius of gyration (Rg), Solvent accessible surface area (SASA) and Hydrogen bond interactions were calculated and plotted utilize Xmgrace (https://plasma-gate.weizmann.ac.il/Grace/) and VMD software^34^.

### Statistical analysis

Statistical analysis was performed using the GraphPad Prism software version 8. Mean ± SD was used to represent the data from this study. Two-way analysis of variance (ANOVA) was used to assess the data and P value is < 0.05 was considered statistically significant. A two-way ANOVA was utilized to assess the impacts of two independent variables and their association on the observed responses. This test was used as it provides the concurrent comparison of several group means while considering interactions among factors, thus minimizing type I error relative to numerous one-way ANOVAs. Post hoc comparisons were conducted utilizing Tukey’s test, which facilitates pairwise group analysis while limiting the family-wise error rate. Prior to performing ANOVA, the presumptions of normality and homogeneity of variances were assessed. The Shapiro-Wilk test was used to evaluate the normality of residuals, whereas Levene’s test was utilized to examine the homogeneity of variances. All data satisfied these assumptions, confirming the suitability of the ANOVA model.

## Results

### Yield of extraction

 The utilization of organic solvents significantly influences the extraction of specific chemical compounds from plant extracts. The importance of polar solvents is to increase the permeability of cell walls for chemical compounds, providing greater interaction between solvent and solute, which consequently elevates the extraction yield percentage. The significant variations in extraction yields are noted between ethanol and other solvents. Table S1 illustrates the percentage efficiency of the crude extracts obtained with different solvents. Among this, the ethanol extract possessed the highest yield (28.2%), followed by chloroform (23.2%) and petroleum ether (4.05%). This study is aligned with previous that the elevated extraction with ethanol is attributable to the solvent’s polarity and as it effectively recovers most polar compounds from various plant extracts. Ethanol was widely employed as a solvent for the separation of phytochemicals from different plant Sect^35^.

### Evaluation of antioxidants with DPPH and FRAP assay

**Fig. 2:**
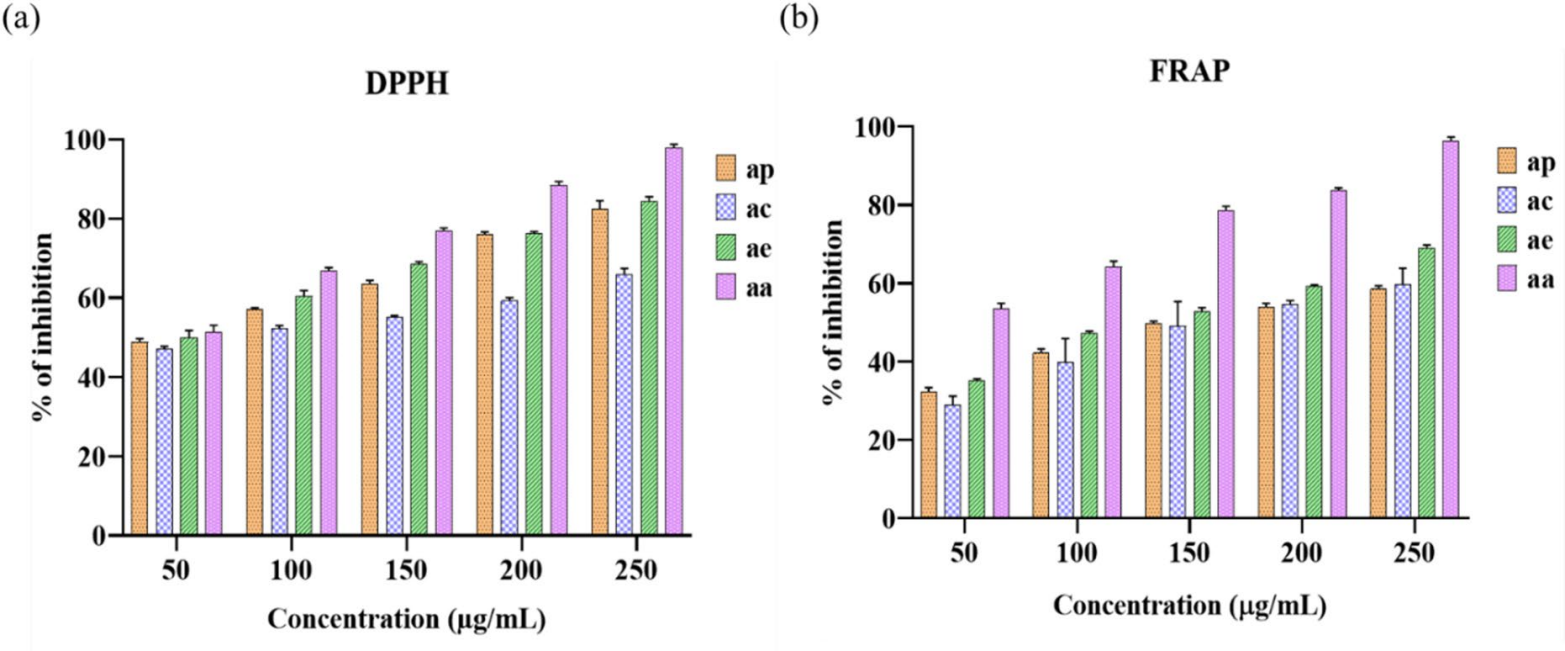
Antioxidant activities of different solvent extracts of *Acalypha indica* at various concentrations compared to ascorbic acid (standard) **(a)** DPPH radical scavenging activity, **(b)** FRAP reducing power assay. *Acalypha indica* petroleum ether extract (ap), *Acalypha indica* chloroform extract (ac), *Acalypha indica* ethanol extract (ae) and ascorbic acid (aa). Data are presented as mean ± SD, (*n* = 3). Statistical analysis was analyzed using two-way ANOVA that follows by Tukey’s post hoc test (*p* < 0.05).

The radical scavenging activities for various solvent extracts of *A. indica* are evaluated by comparing the percentage of DPPH radical production among the different extracts versus ascorbic acid Fig. 2a. In Table 1 illustrates the fact that the DPPH scavenging capacity of different solvent extracts was concentration-dependent (50 µg/mL to 250 µg/mL). An increase in the capacity for DPPH radical is related to a decreased IC_50_ value. IC₅₀ is known as the concentration of an extract desired to inhibit 50% of the free radical’s activities, with a lesser IC₅₀ value signifying higher antioxidant capacity. It was found that the ethanol extract showed higher scavenging effect (84.36%) than the other extracts petroleum ether (82.43%) and chloroform (65.98%) and significantly greater (*P* < 0.05), respectively. In comparison to previous works, the scavenging efficiency of both the methanol and ethanol extracts of *C. ladaniferus* was nearly comparable to that of trolox and ascorbic acid. Extracts from *C. ladaniferus* display the mean inhibitory values of revealing substantial antioxidant activity and suggesting significant possibilities for the process of development of nutraceutical products or addition in the food industry^36^.

**Table 1:** DPPH scavenging activity, the data shown as mean ± SD from three replicates, along with the corresponding IC_50_ values.

DPPH assay				
Concentration (µg/ml)	Petroleum ether	Chloroform	Ethanol	Standard (Ascorbic acid)
50	48.90 ± 0.68	47.23±0.47	50.08± 1.35	51.24± 1.79
100	56.96± 0.42	52.15± 0.67	60.39± 1.15	66.83± 0.76
150	63.5± 0.72	55.2± 0.22	68.64± 0.39	76.94± 0.67
200	75.97± 0.57	59.23± 0.65	76.13± 0.50	88.38± 0.98
250	82.43± 1.74	65.98± 1.17	84.36± 0.93	97.97± 0.80
IC_50_ values	152.7	161.3	143.6	139.5

Data are presented as mean ± SD, (*n* = 3). Statistical analysis was executed using two-way ANOVA that follows by Tukey’s post hoc test (*p* < 0.05).

The abundance of reduction compounds in extracts inhibits free radical chain reactions by donating an atom of hydrogen and thereby lowering Fe3 + ions to Fe2+. The reducing capabilities are frequently correlated with the existence of reductones through demonstrating antioxidant activity by interrupting the free radical chain by hydrogen atom donation. Reductions have been shown for interaction with specific peroxide components and hence inhibiting peroxide development. Figure 2b represents the concentration-response charts indicating the reducing power of different solvent extracts and ascorbic acid. The ferric reducing power shows the percentage of inhibition of different solvent extracts at different concentrations as illustrated in Table 2. Among various extracts, ethanol extract exhibited the highest reducing capacity (69%) while chloroform (59.63%) and petroleum ether (58.46%) were significantly greater (*P* < 0.05), respectively.

**Table 2:** FRAP reducing power, the data shown as mean ± SD from three replicates, along with the corresponding IC_50_ values.

FRAP assay				
Concentration (µg/ml)	Petroleum ether	Chloroform	Ethanol	Standard (Ascorbic acid)
50	32.38 ± 0.83	29.09± 1.75	35.16±0.38	53.45± 1.37
100	42.27± 0.83	39.95± 4.92	47.15± 0.48	64.16± 1.48
150	49.75± 0.43	49.03± 5.11	52.85± 0.72	78.63± 1.05
200	53.84± 0.80	54.58± 0.77	59.28± 0.25	83.60± 0.75
250	58.46± 0.70	59.63± 3.41	69± 0.61	96.42± 0.93
IC_50_ values	281.2	271.8	175.2	161.4

Data are presented as mean ± SD, (*n* = 3). Statistical analysis was executed using two-way ANOVA that follows by Tukey’s post hoc test (*p* < 0.05).

A two-way ANOVA was conducted to assess the antioxidant capacity of *A. indica* extracts (petroleum ether, chloroform, and ethanol) utilizing DPPH and FRAP tests. The findings indicated substantial impacts of extract type and concentration on antioxidant activity in both experiments. The DPPH experiment revealed a significant interaction between extract and concentration (F (12, 40) = 58.30, *p* < 0.0001), exhibiting a substantial effect size (η² = 0.067, partial η² = 0.946) and a confidence level of 99.99%. In the FRAP assay, the interaction effect was significant (F (12, 40) = 5.077, *p* < 0.0001), with an effect size of (η² = 0.020, partial η² = 0.604) and a confidence level of 99.99%. The ethanol extract showed the increased level of antioxidant activity in both DPPH and FRAP experiments, succeeded by the petroleum ether and chloroform, suggesting that increased extract polarity enhances antioxidant capacity. As compared to the previous results, the essential reducing capacity of *L. sativum* extracts is potentially due to specific secondary metabolites that have functionality similar to reductones by providing electrons to free radicals and thereby reducing these molecules into more stable compounds^37^.

### Detection of antimicrobial assay

**Fig. 3:**
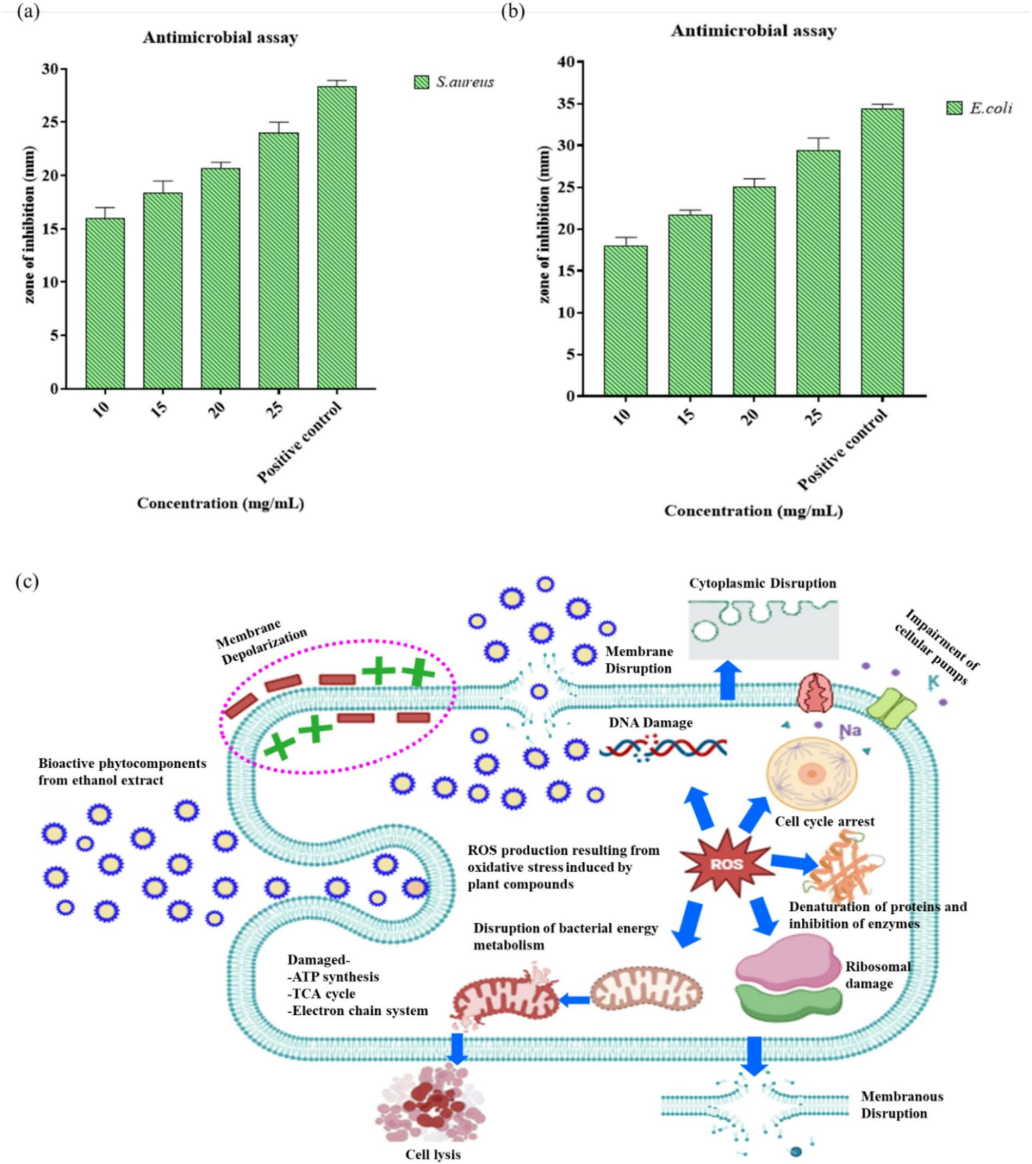
Antimicrobial activity of ethanol extract against food-borne pathogens (**a)** *S. aureus* (Gram positive) and **(b)** *E. coli* (Gram negative). Data are presented as mean ± SD (*n* = 3). Statistical analysis was executed using two-way ANOVA, that follows Tukey’s post hoc test (*p* < 0.05). **(c)** Proposed mechanism of antimicrobial action of bioactive ethanol extract against bacterial cells.

Antibacterial effects of *A. indica* of different solvent extracts against food-borne pathogens and classifies the microorganisms as resistant, intermediate, or susceptible and is widely employed to determine the antimicrobial effectiveness of plant extracts^18^. In Fig. 3a and b represents the results of experiments conducted on various bacteria to determine the antibacterial capability of different solvent extracts. The investigation of the antibacterial effectiveness of these plant extracts indicates the zone of inhibition against foodborne pathogens presented in Table 3. The measurement of the growth inhibition zone ranged from 16 to 28 mm for *S. aureus* and 18 to 34 mm for *E. coli*, as shown at different concentrations. It indicated that ethanol extract was more effective at reducing the tested microbes because the inhibition zones were greater compared to the petroleum ether and chloroform extracts exhibited no/less antibacterial activity. The zone of inhibition represents the lowest concentration of an antimicrobial drug (plant extract) that is required to prevent the growth of microbes. The microorganism’s sensitivity or resistance to the plant extract is determined by comparing the sizes of the zone of interruption with known sizes.

**Table 3:** Antimicrobial activity (zone of inhibition) detected by different solvent extracts against food-borne pathogens and values represented as mean ± SD.

Diameter of inhibition zone (mm)							
	S. aureus				E. coli		
S. No	Concentration (mg/ml)	Petroleum ether	Chloroform	Ethanol	Petroleum ether	Chloroform	Ethanol
1.	10	-	-	16 ± 0.81	-	-	18 ± 1
2.	15	-	-	18.33 ± 0.94	-	-	21.66 ± 0.57
3.	20	-	-	20.66 ± 0.47	-	-	25 ± 1
4.	25	-	-	24 ± 0.81	-	-	29.3 ± 1.52
5.	Tetracycline (positive control)	22.33 ± 0.47	24 ± 0.81	28.33 ± 0.47	23.33 ± 0.47	22 ± 0.81	34.33 ± 0.57
6.	DMSO (negative control)	-	-	-	-	-	-

Data are presented as mean ± SD, (*n* = 3). Statistical analysis was executed using two-way ANOVA that follows by Tukey’s post hoc test (*p* < 0.05).

The ethanol extract demonstrated concentration-dependent antibacterial efficacy against *S. aureus* and *E. coli*. Two-way ANOVA demonstrated significant effects of extract concentration (F (4, 20) = 210.5, *p* < 0.0001), η² = 0.89, 95% CI [0.72–0.94]) and microorganism type (F (1, 20) = 147.0, *p* < 0.0001), η² = 0.88, 95% CI [0.70–0.93]) on the diameter of the inhibitory zone. A substantial interaction between concentration and microbe was noted (F (4, 20) = 4.22, *p* = 0.0122), η² = 0.46, 95% CI [0.18–0.67]). The post-hoc Tukey’s test revealed that the 25 mg/mL ethanol extract shown significantly greater inhibition compared to lower concentrations against both *S. aureus* and *E. coli*. In Fig. 3(c) the bioactive substances present in the plant ethanol extract can interact with the bacterial cell membrane, causing structural damage and increased permeability. These compounds may also penetrate the cytoplasm, leading to the generation of reactive oxygen species (ROS) that DNA damage, enzymes, and proteins. Furthermore, the suppression of essential metabolic processes, including ATP synthesis and the electron transport chain, results in energy depletion, cell lysis and ultimately, bacterial death^38,39^. Based on these findings, *A. indica* might be an efficient way to find wide-ranging antimicrobials. As predicted, the results of this study confirmed that extract of the leaves of *A. indica* was successful at suppressing the growth of the microorganism and utilized for the test and the level of activity was different. Compared to the previous results, the ethanol extract of clove was found to consist of inhibitory zones ranging from 13.4 to 26.3 mm that may eliminate *L. monocytogenes*,* S. aureus*,* V. parahaemolyticus*,* pseudomanads*,* aeromonads and A. faecalis*^40^.

### Determination of antibiofilm assay

**Fig. 4:**
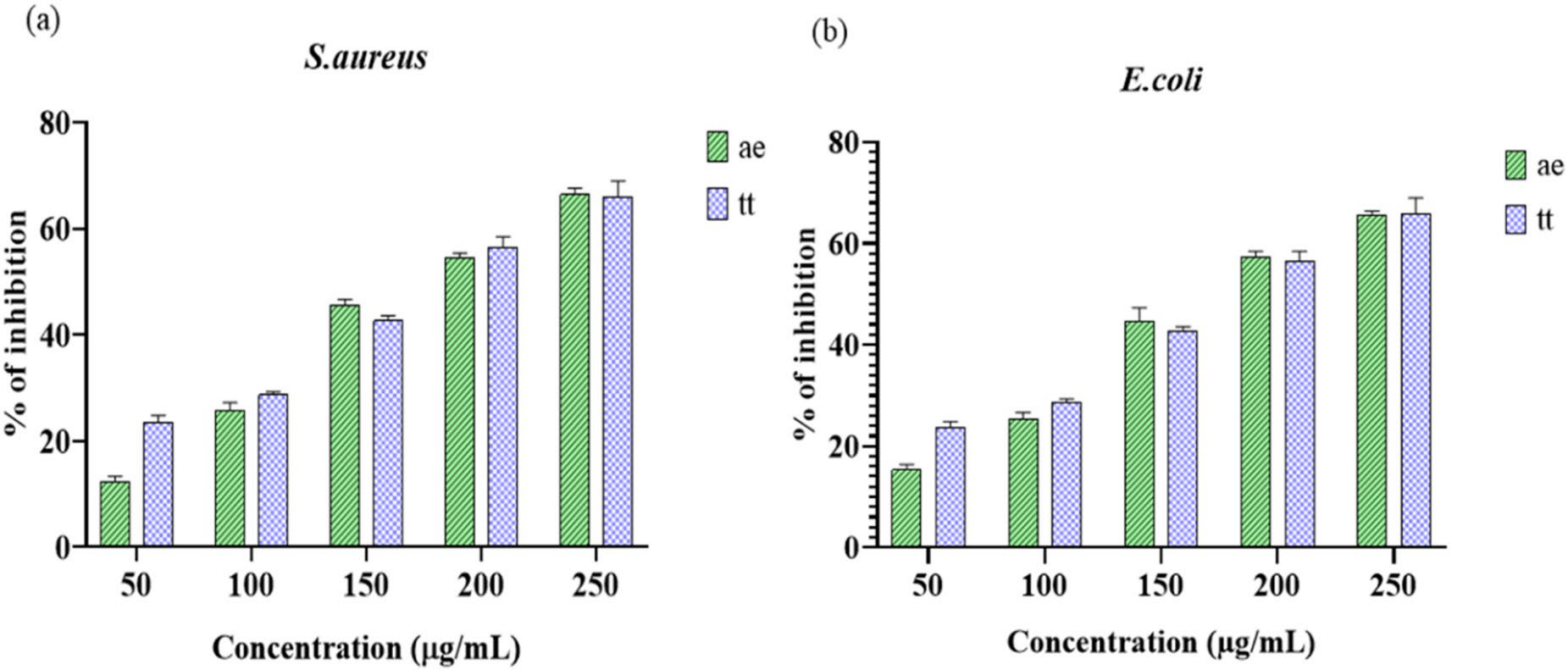
Antibiofilm activity of ethanol extract against food-borne pathogens: (a) *S. aureus* (Gram-positive) and (b) *E. coli* (Gram-negative). *Acalypha indica* ethanol extract (ae) and tetracycline (tt). Data are presented as mean ± SD, (*n* = 3). Statistical analysis was analysed using two-way ANOVA that follows by Tukey’s post hoc test (*p* < 0.05).

Biofilms are layers formed through an adhesion of molecules, including polysaccharides, nucleic acids, sugars and proteins, adhered to living or non-living surfaces. Bacterial biofilms are populations of bacteria that attach to a solid surface and are enclosed in a complex extracellular polymeric matrix. Biofilms accelerate bacterial growth and dispersion with developing antibiotic resistance^41^. In Fig. 4a & b shows the percentage of biofilm inhibition on selected active ethanol extract against food-borne pathogens. In Table 4 depicts an analysis of ethanol extract on adherence and disruption of the development of biofilm. The percentage of inhibition ranges from (12.41 to 66.36%) for *S. aureus* and *E. coli* (15.32 to 65.45%), respectively. A two-way ANOVA was performed to assess the antibiofilm effectiveness of the test samples against *S. aureus* and *E. coli*. The results indicated significant effects of treatment and concentration on biofilm inhibition in both organisms. For *S. aureus*, the interaction effect was significant (F (4,20) = 19.35, *p* < 0.0001), with a large effect size (η² = 0.018, partial η² = 0.795) and a confidence interval (CL) of 99.95%. Similarly, the interaction between treatment and concentration for *E. coli* was statistically significant (F (4,20) = 9.273, *p* = 0.0002), with a large effect size (η² = 0.011, partial η² = 0.649) and a CL of 98.4%. These results indicate that the evaluated treatments significantly reduced biofilm formation in both organisms, demonstrating strong antibiofilm efficacy.

**Table 4:** Antibiofilm Inhibition assay shows the data are given as a mean ± SD of three replicates and IC_50_ values are also mentioned.

% of inhibition			
Concentration µg/ml	S. aureus	E. coli	Tetracycline
50	12.41 ± 0.80	15.32 ± 0.87	23.60 ± 0.98
100	25.84 ± 1.13	25.33 ± 1.03	28.68 ± 0.49
150	45.57 ± 0.89	44.71 ± 2.08	42.66 ± 0.77
200	54.53 ± 0.71	57.23 ± 0.98	56.52 ± 1.57
250	66.36 ± 1.00	65.45 ± 0.74	65.93 ± 2.47
IC_50_ values	195.7	189.8	112.2

Data are presented as mean ± SD, (*n* = 3). Statistical analysis was executed using two-way ANOVA that follows by Tukey’s post hoc test (*p* < 0.05).

Developed criteria imply that percentage values of inhibition ranging from 0 to 100% highlight biofilm inhibition, whilst values below 0% signify growth supplementation. Productivity is regarded as beneficial above the 50% inhibition threshold analysis, whereas it is considered inadequate if the inhibition level decreases between 0 and 49%. The benefit of the plant compounds on exhibit in delaying dependence varies. The findings, particularly those that ranged from 8.99 to 88.3, indicated that plant extracts inhibited the formation of biofilms against *P. aeruginosa*. The inclusion of *S. aureus* and *P. aeruginosa* in the antibiofilm study was justified by their remarkably significant biofilm-forming capacities. *S. aureus* and *E. faecalis* adhesion was successfully inhibited by a variety of plant extracts^42^.

### Response surface methodology

#### Analysis of the model

 In comparison to conventional individual parameter optimization, response surface methodology provides more advantages. Table S2 represents the coded and uncoded independent variables involved in the RSM design. The BBD is utilized for both observed responses and independent variables. The BBD of three variables with their measured values of responses was shown in Table S3. Experiments were performed according to the BBD requiring a total of 15 runs with the **t**hree independent variables and the corresponding values were assigned for RSM optimization in this research study time (30, 45, 60 min), sample concentration (50, 150 and 250 µg/mL) and percentage of ethanol (50, 70, and 90%). Three-dimensional (3D) response surface curves were elevated to assess the interaction among the independent factors and to ascertain the optimum level of each variable for maximal response. It provides a technique to illustrate the correlation between response and experimental levels of each variable along with the type of interactions among components^43^.

#### Analysis of antioxidant activity in optimized conditions (DPPH and FRAP) assay

**Fig. 5:**
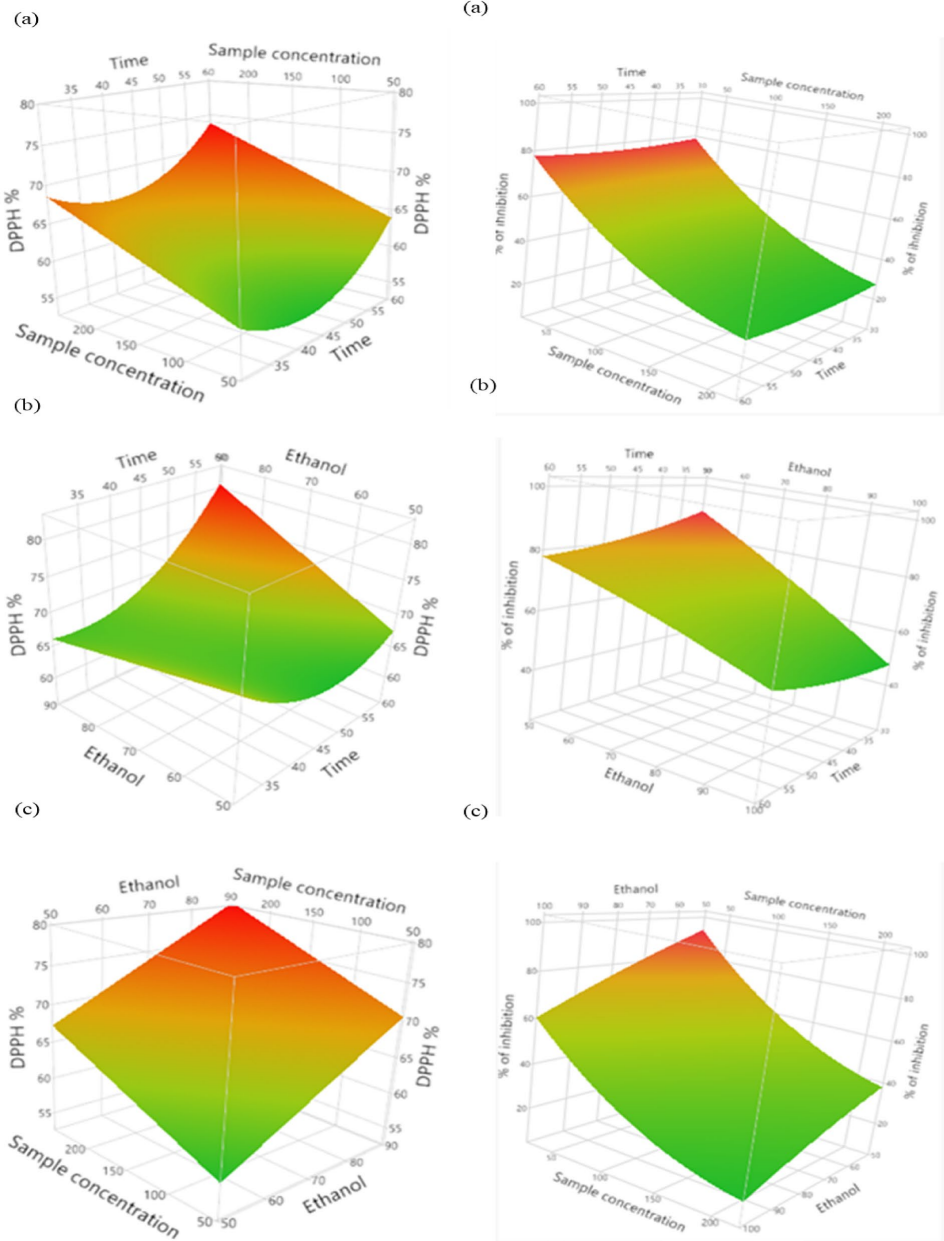
The three-dimensional plot showing the effects of the interaction **(a)** DPPH (a-c) embedded with time, sample concentration and ethanol and **(b)** FRAP (a-c) includes time, sample concentration and ethanol.

 Every response was determined to be associated with the independent variables’ linear, quadratic and interaction^44^. Figure 5(a) DPPH (a) indicates time versus sample concentration (b) involves time versus percentage of solvent and (c) includes solvent versus sample concentration. Figure 5(b) FRAP (a) involves the timings versus sample at different concentrations (b) includes time versus percentage of solvent and (c) examines with sample concentration versus percentage of solvent and explores the three-dimensional plot showing the effects of the interactions and standardized effects of the independent experimental variables. The interactions between the three independent variables were substantial, and the fit of the model sufficiently represented these variables, implying that the second-order polynomial models were appropriately described by the corresponding measurements^43^. It is clear from the obtained equations that the linear model, i.e., X_1_, X_2_, X_3_, and the quadratic model, X_1_^2^, X_2_^2^, X_3_^2^, and the interaction model between all three variables, i.e., X_1_ × _2_, X_1_ × _3_, had a significant effect on the DPPH (0.03) and FRAP (0.001) activity of ethanol extract at different levels (*p* < 0.0001 to *p* < 0.05). The coefficients of multiple determinations R^2^ of 0.91 and 0.96 were obtained for the response of DPPH and FRAP activity, respectively. It shows a strong correlation with the experimental data and lower variance approximately the mean value. RSM was utilized the analysis of variance (ANOVA) of the quadratic models for DPPH and FRAP was represented in Table S4. The findings align with the results reported by Wong et al. (2015) and Pompeu et al. (2009), which concluded that the antioxidant activity is significantly influenced by solvent concentration. The R² value for DPPH scavenging ability was 0.8937, and a lack of fit was extremely non-significant (0.4220), confirming the model’s good fit^45,46^. As compared to the preliminary evaluations performed using various solvents, examine the effectiveness of ethanol in recovering phenolics from *Piper Betle*. Three independent variables and the corresponding values were assigned for RSM optimization in this research study: temperature (50, 60, and 70 °C), ethanol concentration (70, 80, and 90%), and solute-to-solvent ratio (1:10, 1:20, and 1:30 g/mL). The properties and the appropriate parameters evaluated originate from the traditional extraction methods of maceration and Soxhlet^20^.

#### Experimental verification of optimum conditions

To verify the optimal circumstances, the experimental findings were assessed in relation to the ones estimated by the equation using the percentile coefficient of variation (CV). It shows the root mean square error of the antioxidant assay as displayed in Table S5. In comparison to the coefficient of variation, the projected ideal and actual results were 7.4% for antioxidant efficacy, 6.7% for FRAP, and 9.3% for TPC, each of which is deemed low within the target range of optimized outcomes. These findings demonstrate that RSM is a reliable, effective, and legitimate multivariable method for concurrently optimizing numerous variable responses^47^.

### Quantitative analysis for TPC and TFC

**Fig. 6:**
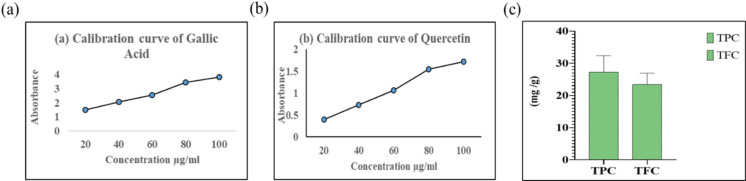
(**a)** Standard curve of Gallic acid to estimate total phenolic content, **(b)** Standard curve of Quercetin to estimate total flavonoid content and **(c)** Ethanol extract to estimate TPC (Total phenolic contents) and TFC (Total flavonoid contents).

The Folin-Ciocalteu reagent was used to measure the ethanol extract of TPC and gallic acid functioning as the standard. Accurate measurement of the phenolic compounds in the extract is rendered attainable by the calibration curve in Fig. 6a, it displays a linear relationship between absorbance and gallic acid concentrations (20–100 µg/mL). Milligrams of gallic acid equivalents (GAE) per gram of extract (mg GAE/g) are used for recording the results (average value 28.24 mg GAE/g). While phenolic compounds can contribute hydrogen atoms or electrons, they are known to have antioxidant properties that effectively neutralize free radicals^48^. Quercetin was used as the standard in the aluminium chloride procedure to determine the TFC. The effectiveness of these techniques for detecting flavonoids is confirmed for the calibration curve for quercetin illustrated in Fig. 6b and displays a linear association between absorbance and quercetin concentrations (20–100 µg/mL). Quercetin equivalents (QE) per gram of extract (mg QE/g) are employed to quantify the results (average value 23.40 mg QE/g). In Fig. 6c displays the TPC and TFC of ethanol extract. Flavonoids contain substantial antibacterial, anti-inflammatory and antioxidant properties and are essential for plant defense systems^49^. According to the quantitative investigation, the ethanol extract includes a greater number of flavonoids and phenolics to especially improve its pharmacological and antioxidant qualities. The absorbance of extract readings against the calibration curves and certain standard methods to calculate their equivalents allows one to ascertain accurate quantities. By calculating the concentration of identified substances, such as the total phenolic and flavonoid content, quantitative analysis might improve these results. Phenols and flavonoids are important because of their widely recognized antibacterial and antioxidant properties.

### TLC analysis

 The ethanol extract was examined using solvents with toluene and ethyl acetate. The optimal separation was achieved at a 4:1 ratio, which indicated areas with R_f_ values. On the TLC sheet, the phytocompounds demonstrated higher R_f_ values, indicating more distance travelled. The data was captured using long ultraviolet light at 365 nm with the findings depicted in Fig. S1 and Table 5. The ethanol extracts comprised a diverse array of phytocompounds.

**Table 5:** TLC analysis of the crude extract using solvents with different ratios.

Crude (Toluene: Ethyl acetate 4:1 ratio)	Fraction (Toluene: Ethyl acetate 4:1 ratio)
0.48	-
0.56	-
0.61	-
0.63	-
0.66	-
0.76	-
0.78	-
0.85	0.72
0.91	-

#### Analysis of antibacterial activity with fractions

**Fig. 7:**
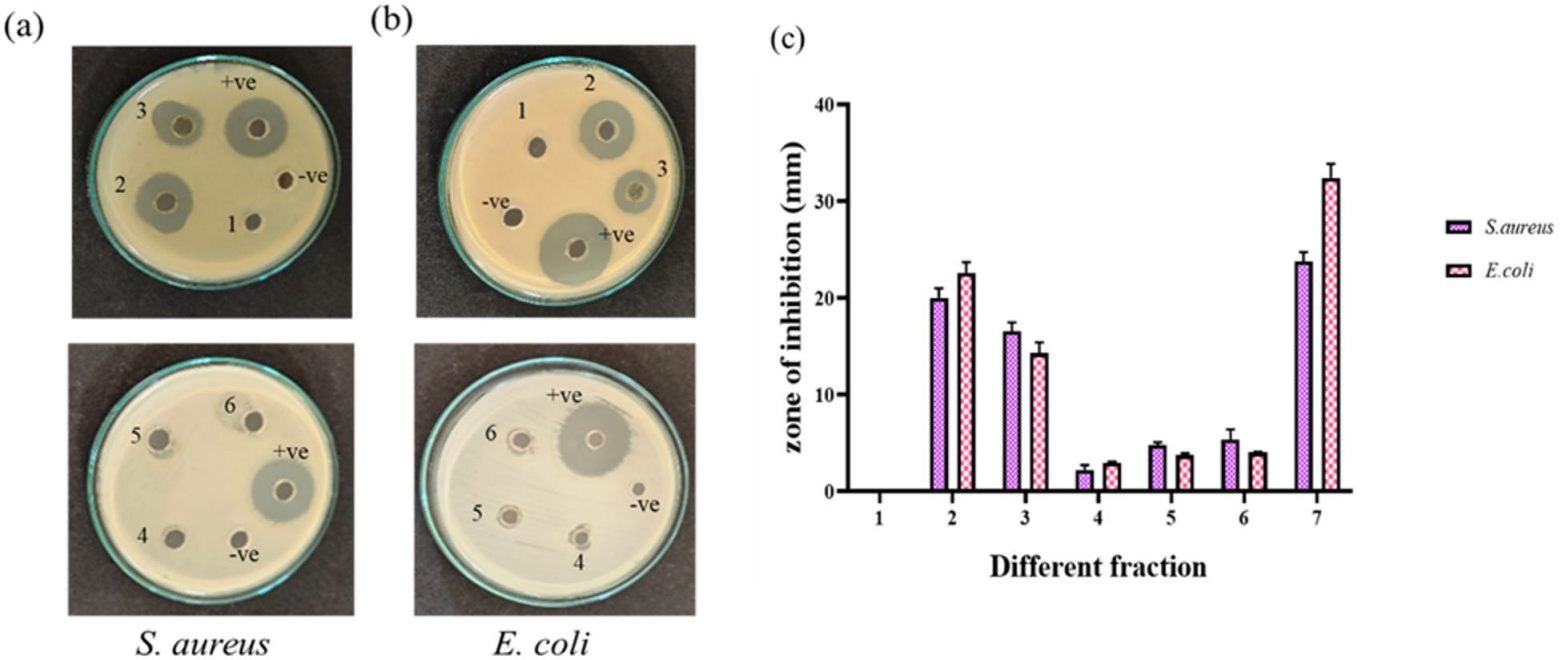
Antimicrobial activity of *Acalypha indica* ethanol extract of six fractions against microorganisms **(a)** *S. aureus*, **(b)** *E. coli* and **(c)** Quantitative results of inhibition growth.

Every portion was methodically acquired by silica gel column chromatography and examined for the identification of various the compounds using TLC. The emitted fractions were combined according to comparable R_f_ values, and each spot was quantified prior to evaporation to prevent moisture loss using a rotary evaporator. The dehydrated weight of each fraction was assessed. Eluates from 164 fractions were collected and subsequently categorized according to TLC results indicating analogous R_f_ values. The six concentrated fractions were subsequently assigned codes from 1 to 6 and evaluated for antibacterial activity. All fractions exhibited inhibitory growth against *S. aureus* and *E. coli*, except for fraction 1. Figure 7 shows the inhibition of growth with antimicrobial plates analyzed with six fractions of *A. indica* ethanol extract for antibacterial activity against foodborne pathogens. The fractions 2 and 3 demonstrated the greatest antibacterial efficacy relative to other fractions against both gram-positive and gram-negative pathogens. *S. aureus* exhibited inhibition zones of 20 mm and 16 mm, while *E. coli* displayed zones of 22 mm and 14 mm. Portion 7 illustrates that the positive control showed a higher efficacy in growth inhibition, but the negative control exhibited no inhibition in all bioassays. Subsequently, the most active fraction 2, was further investigated for its applicability in food preservation and packaging through the creation of edible films.

### GC - MS analysis with fraction

According to Coteli et al.^50^, the GC-MS consideration remains extremely useful for researching the phytochemistry of plants. The fractions separated by TLC were evaluated for antimicrobial activity, and the most potent fraction underwent GC-MS analysis to determine its chemical components. The GC-MS analysis of the partially purified ethanolic extract of *A. indica* identified eleven phytocompounds, which were matched with the NIST and Wiley libraries (Table 6). Fig. S2 illustrates the chemical structures of the major phytoconstituents present in the extract. Several compounds, such as 2-butenoic acid, 1,2-cyclobutane decarboxylate, trans-, and 1-methylpiperazine, have been previously reported for their antinociceptive, anti-inflammatory, antioxidant and antimicrobial activities, supporting the observed bioactivity of the extract. Additionally, compounds including 2(5 H)-furanone, 5-hydroxy-, 2-pentenoic acid, 2-methyl-2-butenoic acid, and 2-butenoic acid (Z)- have been reported as food additives, indicating their potential safety for food-related applications. In contrast, 3-piperidinol, 1,4-dimethyl-, trans-, and N-(2,2-dichloro-1-hydroxyethyl)−2,2-dimethylpropanamide are newly identified in the partially purified ethanolic extract of *A. indica* in this study. These novel compounds also exhibited notable binding affinities in molecular docking analyses. The specific compounds were chosen for molecular docking and ADMET assessment based on specified criteria. The selection was based on their elevated peak area percentage (showing relative abundance), previously identified antioxidant or antibacterial properties and distinctiveness among *A. indica* extracts. Compounds with 0 area% were categorized as trace/detected just and eliminated from the quantitative evaluation. This methodology ensured the inclusion of both established and potentially novel bioactive compounds in computational evaluations. Overall, the GC-MS results validate the presence of known bio actives and suggest the occurrence of novel compounds with potential pharmacological and food preservative relevance.

**Table 6:** GC-MS compounds analysed with structures, molecular formula and weight and biological activities.

S.no	Compound name	Structure	Molecular formula	Molecular weight	Retention time	Area%	Biological activities	References
1.	2-BUTENOIC ACID, 4-OXO-		C_4_H_4_O_3_	100	1.188	27.964	Antinociceptive activity	^62^
2.	2(5 H)-FURANONE, 5-HYDROXY-		C_4_H_4_O_3_	100	1.294	27.061	Food additives and cosmetics	^63^
3.	2-PENTENOIC ACID		C_5_H_8_O_2_	100	1.369	21.449	To analyse the sensory and chemical properties	^64^
4.	2-BUTENOIC ACID, 2-METHYL-		C_5_ H_8_ O_2_	100	1.459	13.490	Therapeutic agents	^65^
5.	2-BUTENOIC ACID, 2-METHYL-, (Z)-		C_5_H_8_O_2_	100	1.579	4.065	Biosynthesis of terpenoids	^66^
6.	1-METHYLCYCLO PROPANE CARBOXYLIC ACID		C_5_H_8_O_2_	100	1.654	2.722	Synthesis of Photovoltaic Polymers	^67^
7.	1,2-CYCLO BUTANE DICARBONITRILE, TRANS-		C_6_H_6_N_2_	106	1.709	3.249	Anti-Inflammatory and Antithrombotic Properties	^68^
8.	2(5 H)-FURANONE, 3-CHLORO-5-((DIMETHYLAMINO)METHYL)−4,5-DIMETHYL-		C_9_H_14_ClNO_2_	203	1.429	-	Chemoresistance	^69^
9.	PIPERAZINE, 1-METHYL-		C_5_H_12_N_2_	100	1.404	-	Antioxidant, antimicrobial and antidepressant activities	^70^
10.	3-PIPERIDINOL, 1,4-DIMETHYL-, TRANS-		C_7_H_15_NO	129	1.499	-	-	-
11.	N-(2,2-DICHLORO-1-HYDROXY-ETHYL)−2,2-DIMETHYL-PROPIONAMIDE		C_7_H_13_Cl_2_NO_2_	213	1.354	-	-	-

Our results align with a study that provided the presence of proteins, lipids, alkaloids, phenols, flavonoids, saponins, tannins, and carbohydrates in *T. hamosa* seeds. It is crucial to recognize that plant extracts can contain a number of metabolites, among which, depending on their bioactivity or bioavailability, may normally show activity or efficacy in in vitro tests^51,52^.

### Molecular Docking analysis with antimicrobial target proteins

**Fig. 8:**
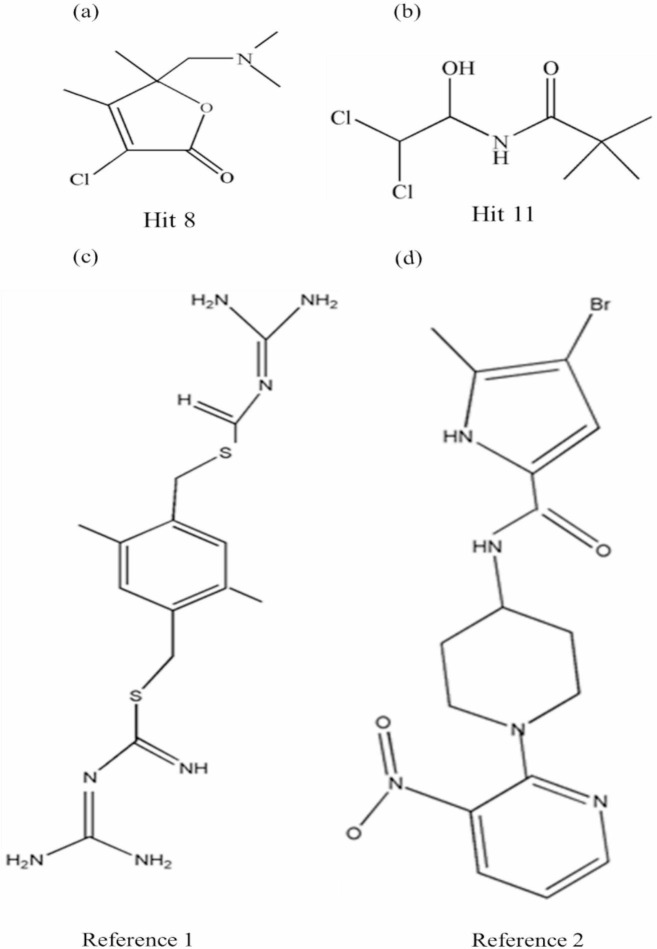
Chemical structures of selected hits and references (**a)** Hit 8, **(b)** Hit 11, **(c)** Reference 1 and **(d)** Reference 2.

**Fig. 9:**
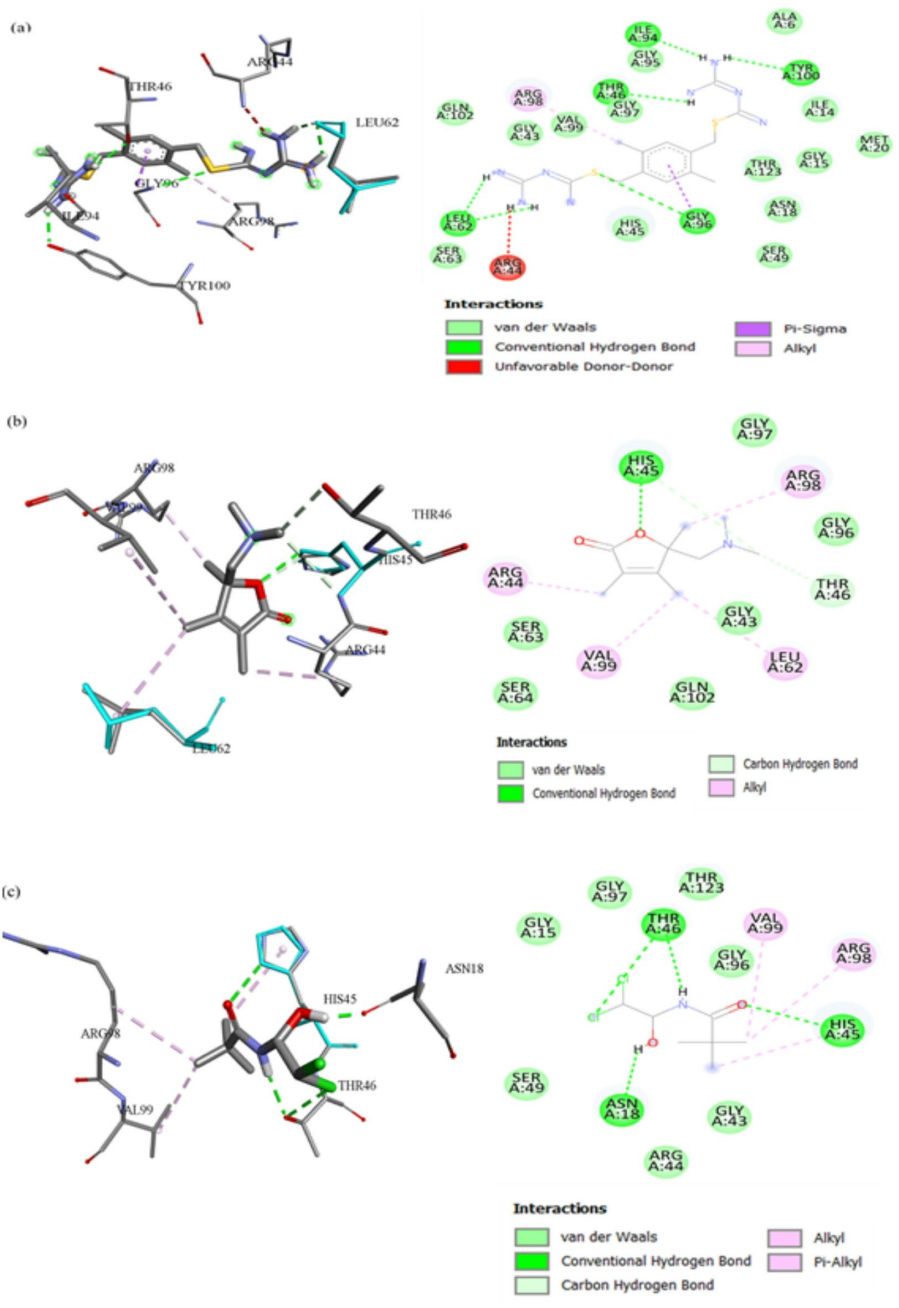
Best-docked structures of GC-MS compounds with critical bacterial protein of GyrB with 3D and 2D (**a)** Reference 1, **(b)** Molecular interaction of Hit 8 and **(c)** Molecular interaction of Hit 11.

**Fig. 10:**
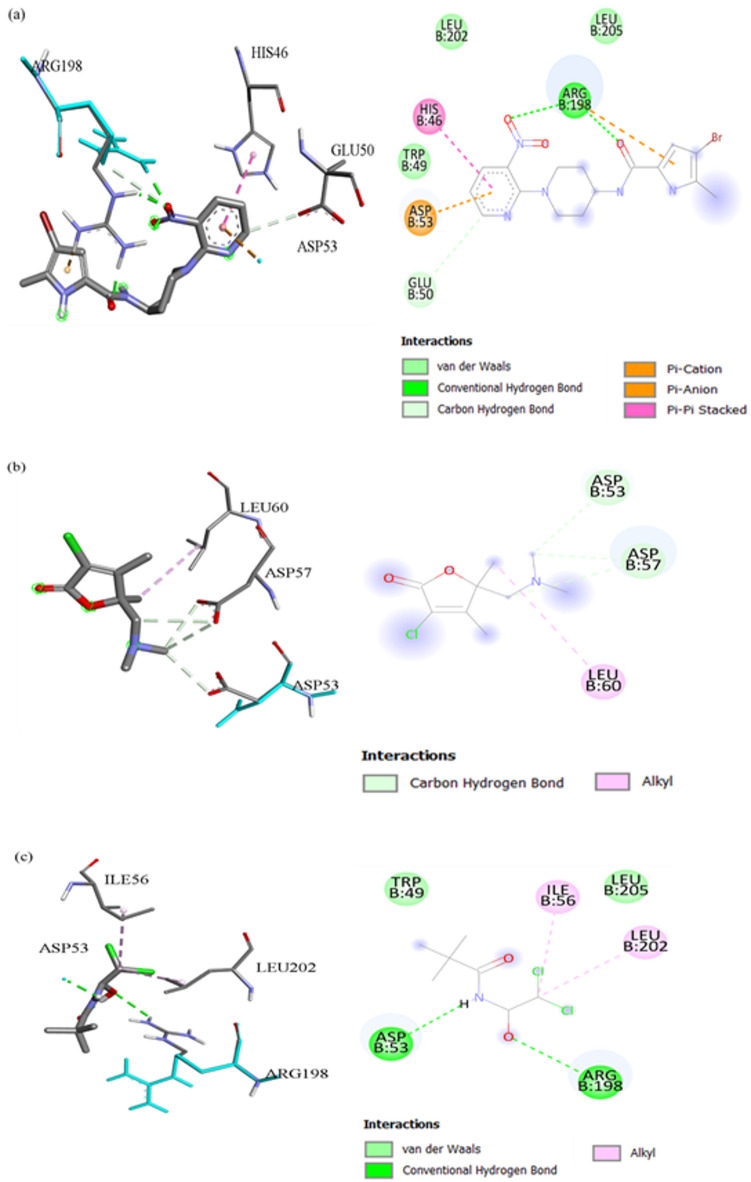
Best-docked structures of GC-MS compounds with critical bacterial protein DFHR with 3D and 2D **(a)** Reference 2, **(b)** Molecular interaction of Hit 8 and **(c)** Molecular interaction of Hit 11.

 The interactions of the compound with target proteins were evaluated by molecular docking to illuminate the mechanism associated with the significant antibacterial activity of the compound. From the GC-MS analysis of 11 phytocompounds were identified, obtained through PubChem in SDF format (https://pubchem.ncbi.nlm.nih.gov/). The retrieved phytocompounds were screened against with the target proteins using Autodock vina along with Swiss ADME/protox and pharmacokinetics properties also analysed. Identified 11 phytocompounds indicated more prominent interactions and passed through ADME filters as Lipinski rule of five shows no violation and analysed with toxicity. The Hit compounds were determined by ADME studies and toxicity results are shown in Table S6 and Table 7. Then, the compounds were subjected to exhaustive docking with 100 iterations, and the results showed the binding energies the ranges from GyrB and DFHR (−3.69 to 6.85 kcal/mol and − 4.28 to −8.75 kcal/mol). One of the compounds (1-Methylpiperazine) displayed the highest binding affinity (6.85 kcal/mol) as compared to reference 1 (GyrB). The docking results of the studies with the target proteins the total number of bonds, interactions of amino acid residues and exhaustive docking score were depicted in Table 8. By Post Docking analysis, the top two compounds (Hit 8 and Hit 11) were selected based on docking score and interacting amino acid residues compared with reference compounds for the respective proteins. Figure 8 illustrates that the chemical structures of selected hits and references Hit 8, Hit 11, Reference 1 and Reference 2. The reference and Hit compounds showed the results of molecular docking interactions in both 2D and 3D visualizations in Figs. 9 and 10. Unfortunately, neither Hit8 nor Hit11 were evaluated for any biological activity, these compounds are yet be explored individually for different assays. Based on *insilico* investigation outcome, we can predict that among all the compound present in the partially extract these two Hits compounds are probably responsible for the activity of extract. Consequently, both the Hit compounds were carried forward for molecular dynamic simulation studies with target proteins. The redocking of the co-crystallized ligand replicated the experimental binding position within an RMSD tolerance of 2.0 Å, thereby validating the reliability of the docking method for further studies. In general, more negative docking scores correspond to stronger binding affinities, indicating more stable ligand-protein interactions^34^.

**Table 7:** Analysis of hit compounds through the toxicity.

Compounds/Control	LD_50_ (mg/kg)	Toxicity Class		Hepatotoxicity	Carcinogenicity	Immunotoxicity	Cytotoxicity	Mutagenicity
Hit 8	1000	4	Prediction	Inactive	Inactive	Inactive	Inactive	Inactive
			Probability	0.61	0.53	0.90	0.71	0.54
Hit 11	1850	4	Prediction	Inactive	Inactive	Inactive	Inactive	Inactive
			Probability	0.71	0.55	0.99	0.64	0.75
Reference 1	120	3	Prediction	Inactive	Inactive	Inactive	Inactive	Inactive
			Probability	0.67	0.62	0.99	0.62	0.65
Reference 2	200	3	Prediction	Inactive	Active	Active	Inactive	Inactive
			Probability	0.54	0.60	0.95	0.56	0.81

**Table 8:** Docking analysis of selected phytocompounds against with the antimicrobial target proteins of *S. aureus* DNA gyrase B (GyrB) and *E. coli* dihydrofolate reductase (DHFR).

S. aureus					E. coli		
**Hits**	**Phytocompounds**	**Exhaustive score (kcal/mol)**	**Conventional H-bonds and Carbon hydrogen bonds**	**Other interactions**	**Exhaustive score (kcal/mol)**	**Conventional H-bonds and Carbon hydrogen bonds**	**Other interactions**
1	2-BUTENOIC ACID, 4-OXO-	−3.82	Asp53, Arg198	VD: His46, Trp49, Glu50, Lys163, Leu202	−4.38	His45, Thr46, Gly96	VD: Gly43, Arg44, Gly95, Arg98, Val99
2	2(5 H)-FURANONE, 5-HYDROXY-	− 3.72	Arg198	VD: Trp49, Lys163, Leu202	−4.28	His45, Thr46, Asn18	VD: Gly43, Arg44, Gly96, Gly97; Alkyl bond: Arg98, Val99
3	2-PENTENOIC ACID	−4.18	Asp53, Arg198	VD: trp49, Glu50, Lys163, Leu202	−4.81	Arg44, Leu62	VD: Gly43, Thr46, Ser63, Gly96, Arg98; Alkyl bond: His45, Val99
4	2-BUTENOIC ACID, 2-METHYL-	−4.15	Asp53, Arg193	VD: Trp49, Glu50, Lys163	−5.01	Glu101	VD: Arg12, Gly97, Arg98, Tyr100, His124, Phe125; Alkyl bond: Pro126
5	2-BUTENOIC ACID, 2-METHYL-, (Z)-	−3.75	Asp53, Arg198	VD: Lys163, Leu202; Alkyl bond: His46, Trp49	−4.94	Arg98, Thr123	VD: Arg12, Gly97, Tyr100, His124, Phe125, Pro126, Asp127; Alkyl bond: Glu101
6	1-METHYLCYCLO PROPANE CARBOXYLIC ACID	−3.69	Asp53, Arg198	VD: Lys163, Leu202; Alkyl bond: Trp49	−4.75	Glu101	VD: Gly97, Arg98, Tyr100, Thr123, His124; Alkyl bond: Pro126
7	1,2-CYCLO BUTANE DICARBONITRILE, TRANS-	−4.4	His46, Arg198	VD: Arg42, Lys163, Leu202; Alkyl bond: Trp49	−6.77	His45, Thr46, Asn18	VD: Gly43, Arg44, Gly96, Gly97; Alkyl bond: Arg98, Val99
**8**	**2(5 H)-FURANONE**, **3-CHLORO-5-((DIMETHYLAMINO)METHYL)−4**,**5-DIMETHYL-**	**−5.21**	**Glu164**	**VD: Asp53**,** Leu162; Alkyl bond: Leu60**,** Lys163**	**−8.38**	**His45**	**VD: Gly43**,** Ser63**,** Ser64**,** Gly96**,** Gly97**,** Gln102; Alkyl bond: Arg44**,** Leu62**,** Arg98**,** Val99**
9	PIPERAZINE, 1-METHYL-	−6.85	Glu50	VD: Asp53, Asn54, Asp57	−4.94	Thr46	VD: Gly43, Arg44, His45, Gly96, Gly97, Arg98, Val99
10	3-PIPERIDINOL, 1,4-DIMETHYL-, TRANS-	−5.25	Asp53	VD: Glu50, Asn54; Alkyl bond: Lys163	−6.55	Thr46	VD: Gly43, Arg44, Trp47, Gly96, Gly97, Thr123; Alkyl bond: His45, Arg98, Val99
**11**	**N-(2**,**2-DICHLORO-1-HYDROXY-ETHYL)−2**,**2-DIMETHYL-PROPIONAMIDE**	**−4.39**	**Asp53**,** Lys163**,** Arg198**	**VD: Trp49**,** Glu164**,** Leu205; Alkyl bond: Ile56**,** Leu202**	**−8.75**	**Asn18**,** His45**,** Thr46**	**VD: Gly15**,** Gly43**,** Arg44**,** Ser49**,** Gly96**,** Gly97**,** Thr123; Alkyl bond: Arg98**,** Val99**
**Reference 1**	**2**,**5-DIMETHYLBENZENE-1**,**4-DIYL) DIMETHANEDIYL BIS (N-CARBAMIMIDOYLCARBAMIMIDOTHIOATE**	**−13.27**	**Thr46**,** Leu62**,** Ile94**,** Gly96**,** Tyr100**	**VD: Ala6**,** Ile14**,** Gly15**,** Asn18**,** Met20**,** Gly43**,** His 45**,** Ser49**,** Ser63**,** Gly95**,** Gly97**,** Val99 Gln102**,** Thr123; Alkyl bond: Arg98**,** Val99**	**-**	**-**	**-**
**Reference 2**	**4-BROMO 5-METHYL-N-(1-(3-NITROPYRIDIN-2-YL) PIPERIDIN-4-YL)−1 H-PYRROLE-2-CARBOXAMIDE**	**-**	**-**	**-**	**−6.3**	**Tyr141**,** Lys163**,** Arg198**,** Glu50**	**VD: Trp49**,** His150**,** Phe160**,** Val165**,** Leu202**,** Leu205; Alkyl bond: His46**

* VD- Vander Waals.

### Molecular dynamic simulations (MDS)

After exploring with post docking studies of Hit compounds and MDS was utilized for evaluating the stability, behaviour, binding approach and imparts to more understanding for protein ligand complexes^53^. The top two docked ligands (Hit 8 and 11) complexed with References 1 and 2 were performed for 100 ns and the results were examined. Furthermore, the MD simulation trajectory assesses RMSD, RMSF, Rg, SASA and the number of bonds containing hydrogen to examine the conformational characteristics of the receptor-ligand complex, encompassing both stability and versatility.

#### Root mean square deviation (RMSD)

**Fig. 11:**
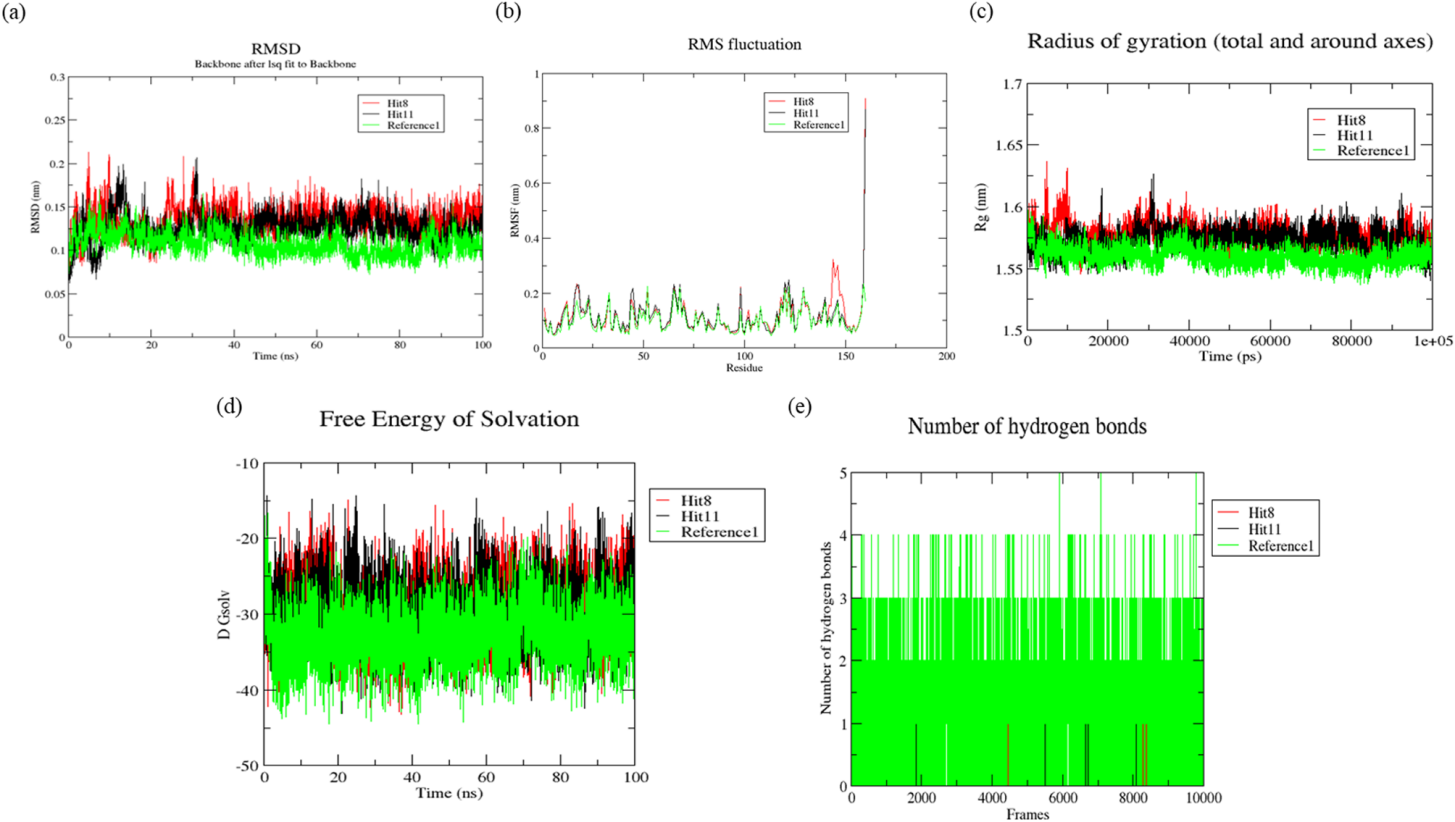
Molecular dynamic simulation trajectory analysis of GyrB protein and protein-ligand complexes during 100 ns simulation **(a)** RMSD plots of Hit 8, Hit 11 and Reference 1, **(b)** RMSF plots of Hit 8, Hit 11 and Reference 1, **(c)** Rg plots of Hit 8, Hit 11 and Reference 1, **(d)** SASA plots of Hit 8, Hit 11and Reference 1and **(e)** Hydrogen bonds plots of Hit 8, Hit 11 and Reference 1.

**Fig. 12:**
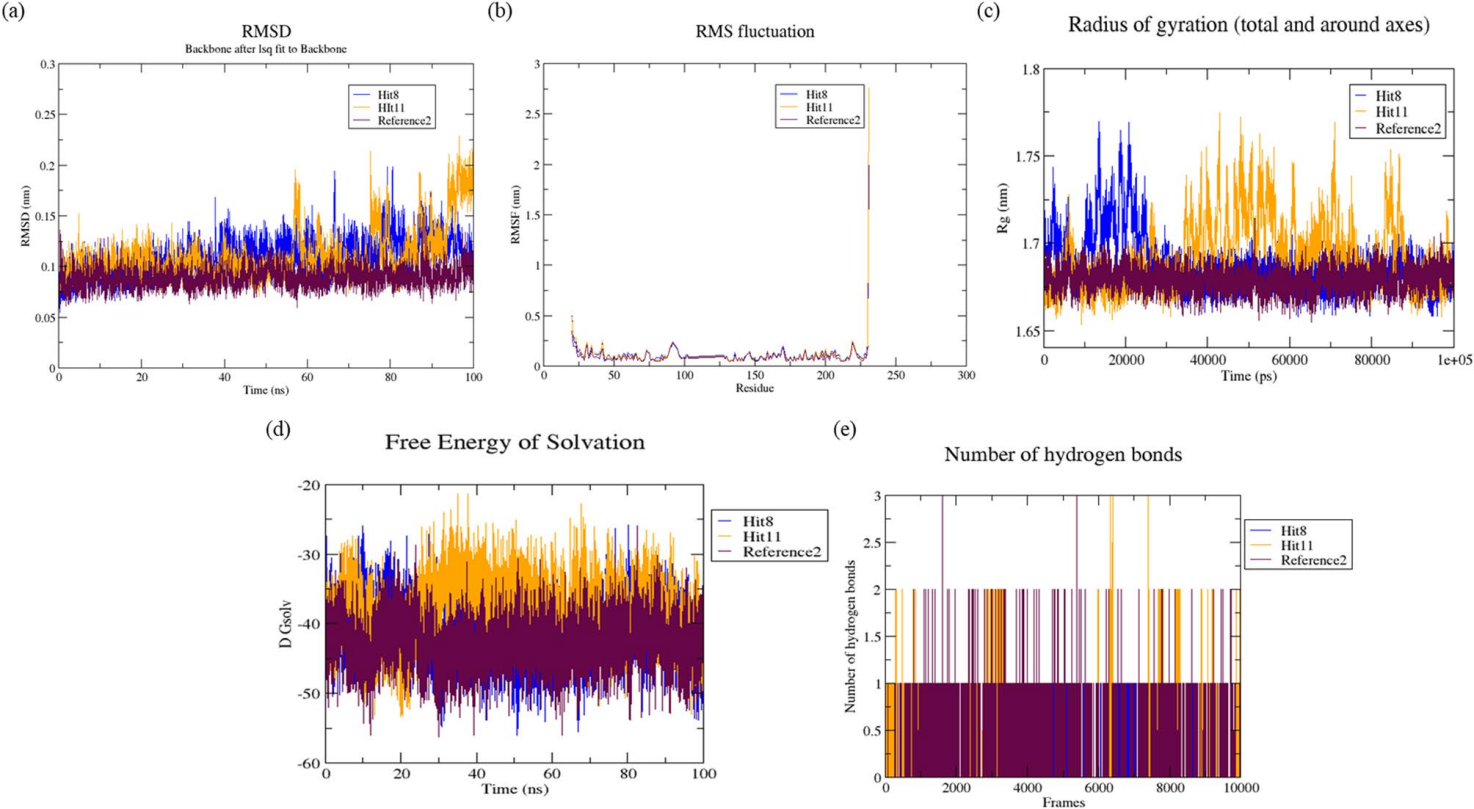
Molecular dynamic simulation trajectory analysis of DFHR protein and protein-ligand complexes during 100 ns simulation **(a)** RMSD plots of Hit 8, Hit 11 and Reference 2, **(b)** RMSF plots of Hit 8, Hit 11 and Reference 2, **(c)** Rg plots of Hit 8, Hit 11 and Reference 2, **(d)** SASA plots of Hit 8, Hit 11 and Reference 2 and **(e)** Hydrogen bonds plots of Hit 8, Hit 11 and Reference 2.

The average values constitute for estimate of the reliability of the receptor-ligand interaction and flexibility. The yields crucial understanding into protein structure, dynamics and function, facilitating the comprehension of biological processes and the formulation of therapeutic methods^54^. RMSD depiction of protein complexes with GyrB and DFHR is shown in Figs. 11a and 12a. The RMSD average values for all docked complexes with proteins have been determined in Table 9. In the GyrB protein, the Hits 8 and 11 both showed similar trajectory. Similarly, in the DFHR protein, the Hits 8 and 11 both indicated similar trend of trajectory throughout the simulations. RMSD plots signified the stability of both Hits comparatively stable to the respective reference compound.

**Table 9:** Represents the average values of hits compounds against with target proteins.

GyrB				DFHR		
	Hit 8	Hit 11	Reference 1	Hit 8	Hit 11	Reference 2
RMSD	0.134 ± 0.015	0.125 ± 0.015	0.107 ± 0.013	0.108 ± 0.017	0.110 ± 0.025	0.089 ± 0.010
RMSF	0.119 ± 0.081	0.117 ± 0.076	0.102 ± 0.042	0.121 ± 0.201	0.092 ± 0.045	0.119 ± 0.146
Rg	1.577 ± 0.009	1.572 ± 0.009	1.560 ± 0.007	1.687 ± 0.015	1.692 ± 0.018	1.679 ± 0.006
SASA	−29.302 ± 3.980	−29.370 ± 3.884	−32.629 ± 3.580	−41.163 ± 4.065	−37.887 ± 4.396	−42.873 ± 3.628
Hydrogen bonds	0 to 2	0 to 3	0 to 5	0 to 1	0 to 3	0 to 3

#### Root mean square fluctuation (RMSF)

It is employed as a measurement of the mobility of amino acid residues, and the values signify protein flexibility and structural integrity. A low RMSF (< 0.1 nm) typically indicates stability, stiffness and robust interactions within the protein substances. An elevated RMSF value (> 1.0 nm) indicates significant flexibility, disordered regions and perhaps aggregation^55^. The RMSF of average values for all docked complexes have been examined in Figs. 11b and 12b and depicted in Table 9. It has been indicated that Hits 8 and 11 both have almost similar fluctuations in the GyrB protein. In the case of DFHR, the Hit 11 demonstrated that relatively lower RMSF value compared to the reference 2 and proves that the complex is more stable and stiffens while Hit 8 have showed similar fluctuations.

#### Radius of gyration (Rg)

A significant metric to evaluate the overall size and compactness of macromolecules such as proteins is the Rg. Lower Rg values indicate a more compact, globular structure, whereas larger values reflect an extended or unfolded conformation^34^. It has calculated the average values of Rg based on the inherent dynamics of protein-ligand complexes as shown in Figs. 11c and 12c and examined Table 9. It has been observed that all complexes are more compact with nearly same Rg values signifying the stability throughout the simulations.

#### Solvent accessible surface area (SASA)

It is an essential parameter in molecular dynamics, assessing the surface area of a molecule that is possible for stability. A lower SASA value correlates with increased compactness and stability of the protein^56^. The SASA plots and values has been shown in Figs. 11 d and 12 d and depicted in Table 9. A solvent affects protein-ligand interactions and protein folding. Both the Hits showed lower SASA values than the reference compound on both proteins. This indicated more stability of Hit compounds than the reference compounds on the both the proteins during simulation periods.

#### Hydrogen bonds

Evaluation of hydrogen bonds provides essential insights into molecular interactions, stability and efficiency^57^. During the 100 ns of the receptor-ligand MD simulations, the H-bonds were captured in Figs. 11e and 12e and shown in Table 9. In the case of GyrB proteins, Hits 8 and 11 showed fewer hydrogen bonds. In the case of DFHR protein, Hit 8 showed similar hydrogen bonds and indicated more efficiency throughout the simulations and Hit 11 exhibited fewer hydrogen bonds.

## Discussion

Plant-based preservatives operate by inhibiting microbial growth, reducing oxidation, and extending the shelf life of food products. As consumer awareness of food safety and environmental issues rises, particularly with chemical preservatives and plastic packaging, there is a growing interest in natural antibacterial and antioxidant compounds. Medicinal plants, historically acknowledged for their therapeutic characteristics, offer a significant reservoir of bioactive components with prospective uses in food preservation. Our investigation demonstrated that the extraction technique yielded a substantial quantity of polar molecules, aligning with the solubility of phytoconstituents in polar solvents. These extracts exhibited significant antioxidant capability, as evidenced by DPPH radical scavenging experiments, indicating their capacity to reduce oxidative degradation of dietary constituents like pigments, lipids, and vitamins^58^. The influence of radicals on DPPH is attributed to its electron-donating capability. Plant-derived extracts possessing antioxidants with scavenging capabilities can provide hydrogen to lipid peroxidase or hydro peroxidase free radicals and are considered essential propagators of lipid chain autoxidation. It also generates non-radicals that interact with the lipid peroxidation chain reaction^16^. This assay finds the existence of phenolics substance and flavonoid components in plant extracts and is a frequently employed method for examining the antioxidant properties of these extracts.

Furthermore, to the antioxidant activity, the extracts exhibited significant antibacterial properties. The ethanolic extract of *A. indica* shown potential candidates for use as natural food preservatives against *S. aureus*, a prominent foodborne pathogen, hence corroborating its traditional therapeutic application in infection treatment. The activity can be ascribed to many mechanisms, such as rupture of bacterial membranes, blockage of quorum sensing, and interference with biofilm formation-crucial activities that augment bacterial resistance to preservatives. These findings highlight the combined antioxidant and antibacterial capabilities of plant extracts to combat spoilage and pathogenic microorganisms. The antibiofilm activity exhibited by the partially purified ethanol extract of *A. indica* is especially relevant, considering that biofilm development is an essential factor in microbial resistance. The extract’s ability to impede biofilm formation indicates disruption of cell adhesion or quorum-sensing processes. Comparable antibiofilm effects have been described for additional phytochemical substances, corroborating our results. The results suggest that *A. indica* extract might serve as a natural alternative for controlling biofilm-associated resistant microorganisms and improving food preservation safety^59^.

RSM is a reliable statistical technique for maximizing process factors and serves as an effective tool to identify the best conditions that enrich a process while performed effectively. RSM elucidates the impact of independent variables, whether individually or in combination related to the procedures. It serves as an effective method for optimizing chemical and biological processes compared to the classical one factor at a time strategy but is comparatively time-consuming and costly^47^. In comparison to other research on *A. indica*, our results indicate that the ethanol extract had enhanced antioxidant and antibacterial properties, probably attributed to its elevated levels of phenolic and flavonoid components. Ethanol efficiently extracts these bioactives, that may provide hydrogen or electrons to neutralize free radicals, change bacterial membranes, or inhibit critical enzymes. These findings align with previous studies and emphasize ethanol’s efficacy as a solvent for extracting multifunctional chemicals that contribute to the exhibited biological effects^60^.

Computational methods further validated these findings. Bioactive compounds identified by GC-MS were analyzed using molecular docking against antimicrobial target proteins, demonstrating significant binding affinities and stabilizing interactions, notably hydrogen bonds and hydrophobic contacts^52^. The docking scores were evaluated to determine the binding affinities of the selected compounds toward the target proteins. Docking scores of ≤ −7.0 kcal/mol are typically regarded as indicative of strong binding affinity. In this study, Hit 8 (−8.38 kcal/mol) and Hit 11 (−8.75 kcal/mol) demonstrated docking scores within a favorable range, implying persistent and favorable energetic interactions with the active site residues. Reference 1 (−13.27 kcal/mol) exhibited the highest binding affinity, whereas Hit 8 and Hit 11 displayed higher binding compared to Reference 2 (−6.30 kcal/mol), underscoring their potential as effective inhibitors. The pharmacokinetic and toxicity properties of all drugs were predicted with SwissADME and ProTox-II. The comprehensive ADMET and toxicity data, including molecular weight, LogP, TPSA, gastrointestinal absorption, blood-brain barrier permeability, LD₅₀, and toxicity classification, are consolidated in Table S6. The findings demonstrate that the majority of drugs exhibit favorable pharmacokinetic properties and low acute oral toxicity (toxicity classes 4–6), hence supporting their appropriateness for future development.

We demonstrate that the RMSD, radius of gyration, and secondary structure levels attained stable plateaus following the initial equilibration phase. The block averaging of the final 20–30 ns further confirmed that the thermodynamic and structural parameters were constant across independent time intervals. The results validate that the 100 ns simulations were adequate to reflect the equilibrium actions of our system’s components. ADME predictions further suggested that the lead compounds are non-toxic and potentially appropriate for food-related applications^12^. The reference ligands exhibiting slightly higher docking scores, the Hit 8 and Hit 11 were selected for further study due to their equivalent binding affinities and consistent interaction patterns within the active domain. Both compounds formed significant hydrogen bonds, hydrophobic interactions with essential catalytic residues, closely resembling the binding behavior of the reference ligands. Notwithstanding the slightly lower docking scores, these persistent and specific interactions suggest that Hit 8 and Hit 11 may exhibit favorable biological activity and potential as efficacious inhibitors.

The in vitro activity results often align with the docking predictions, suggesting that compounds with higher binding affinities and more favorable interaction patterns in silico are probable to demonstrate enhanced biological responses experimentally. However, discrepancies between computational and experimental findings highlight the intrinsic limits of docking investigations, which fails to adequately consider protein flexibility, solvent interactions, or cellular variables affecting bioavailability and metabolism. The integrated analysis of docking and in vitro results enhances the comprehension of the structure-activity relationship, with docking delivering mechanistic insights and rational prioritization, while experimental validation substantiates the biological significance of the predicted interactions^61^.

### Limitation of the study

Overall, our findings indicate that the selected hit compounds have multifunctional features, integrating antioxidant activity, antibacterial efficiency, and advantageous binding interactions with bacterial targets. The current investigation demonstrates the potential antioxidant and antimicrobial properties of *A. indica* extracts. Although the current study demonstrated significant antimicrobial activity of the ethanol extract using the agar well diffusion method and a limitation of the current work the minimum inhibitory concentration (MIC) and minimum bactericidal concentration (MBC) were not determined. Future studies will focus on determining MIC and MBC values to provide a more quantitative evaluation of the extract’s antibacterial potential. Nevertheless, further studies are needed to evaluate their cytotoxicity, in vivo animal investigations to ascertain the safety and sensory effects in real food matrices. Such investigations will provide a comprehensive understanding of their safety and practical applicability as natural food preservatives.

## Conclusion

The phytochemical and biological properties of *A. indica* emphasize its significance as a valuable source of bioactive substances. In this current study, the biological potential of partially purified ethanol extract of *A. indica* was analysed using a dual approach that combined experimental assays within silico molecular docking analyses. GC-MS profiling revealed many bioactive compounds, and the comprehensive study indicated that these constituents exhibit significant antimicrobial and antioxidant properties, validating their potential application in food preservation. This work’s novelty consists in utilizing a partially purified ethanol extract rather than crude or isolated compounds, thus integrating existing extraction-based screening with computational validation. It indicates that the extract appeared to be a promising natural preservative with potential applications in agriculture, cosmetic and the food industry, particularly as active packaging (films and coatings). Future work will focus on the isolation of active compounds, in vivo validation, and formulation of packaging films for food preservation.

## Supplementary Information

Below is the link to the electronic supplementary material.

Supplementary Material 1

## Data Availability

Data will be made available upon reasonable request.
