# Supplementary material for: “Therapeutic potential of Acalypha indica L. leaf fractions against foodborne pathogens: an in vitro and in silico study”
Source: Sci Rep. 2025 Dec 19;16:2423. doi: 10.1038/s41598-025-32216-2 (PMC12820239; doi:10.1038/s41598-025-32216-2)
Supplement: Supplementary file 1 — Supplementary Material 1 [file 41598_2025_32216_MOESM1_ESM.docx]

**“Therapeutic potential of *Acalypha indica* L. leaf fractions against foodborne pathogens: An *in vitro* and *in silico* study”**

Uma Venkatesan^1^, Rajiniraja Muniyan*^1^

School of Bio - Sciences and Technology^1^, Vellore Institute of Technology, Vellore 632014,

India

**Table S1** Percentage yield of the extracts obtained from the *A. indica* plant using different solvent

| **Medicinal plant** | **Solvents** | **Initial weight of the sample (g)** | **Weight of the dried extract (g)** | **Yield of extraction (%)** |
| --- | --- | --- | --- | --- |
| *Acalypha indica* | Petroleum ether | 25 | 0.81 | 4.05 |
|  | Chloroform |  | 4.65 | 23.2 |
|  | Ethanol |  | 5.6 | 28.2 |

**Optimization study using Response Surface Methodology (RSM)**

The experimental results for the three responses were adapted to a second-order polynomial model as represented by the following equation:

Y = a_0_+a_1_x_1_+a_2_x_2_+a_11_x_1_^2^+a_22_x_2_^2^+a_12_x_1_x_2_

Where y is the measured response variable x_1_ and x_2_ represent the levels of independent variables, a_0_ is a constant (predicted response at the center) and a_1_, a_2_, a_11_, a_22_ and a_12_ are the linear, quadratic and two-factor interaction coefficients of the model, respectively. Coefficients of determination (R^2^) were calculated. The RSM-derived model was validated by further tests in triplicate under optimal conditions. The root mean square error (RMSE) percentages were used for comparing the predicted values with the experimental results^15^.

Root mean square error (%) = $\frac{Experiment value-predicted value}{predicted value} \times100$

Experimental value = observed value obtained from experiments

Predicted value = value obtained from the model.

The design of the study was executed with JMP 10 software (Statistical Analysis System Inc., SAS). The widespread second-order polynomial emulation used for the response surface analysis is presented. To evaluate the model's integrity and its predicted results were compared with the experimental results.

**Table S2** Independent variable levels in experimental design for response surface analysis

| **Independent variables** | **Units** | **Symbol** | **Coded levels** | | |
| --- | --- | --- | --- | --- | --- |
|  |  |  | **-1** | **0** | **+1** |
| Time | Minutes (mins) | X_1_ | 30 | 45 | 60 |
| Sample concentration | µg/ml | X_2_ | 50 | 150 | 250 |
| Ethanol | ml | X_3_ | 50 | 70 | 90 |

**Table S3** Box-Behnken design of three variables with their measured values of responses

| **Run** | **X_1_: Time** | **X_2:_ Sample concentration** | **X_3_: Ethanol** |
| --- | --- | --- | --- |
| 1 | 45 | 50 | 50 |
| 2 | 45 | 250 | 90 |
| 3 | 45 | 150 | 70 |
| 4 | 30 | 150 | 50 |
| 5 | 30 | 50 | 70 |
| 6 | 45 | 150 | 70 |
| 7 | 30 | 250 | 70 |
| 8 | 45 | 150 | 70 |
| 9 | 30 | 150 | 90 |
| 10 | 60 | 250 | 70 |
| 11 | 45 | 50 | 90 |
| 12 | 60 | 50 | 70 |
| 13 | 60 | 150 | 50 |
| 14 | 60 | 150 | 90 |
| 15 | 45 | 250 | 50 |

#

#

# **Table S4** Analysis of variance (ANOVA) of the quadratic models for DPPH and FRAP

| **Constants** | **DPPH %** | **ABTS %** | **FRAP %** |
| --- | --- | --- | --- |
| X_1_: Time | 0.2473 | 0.66 | 0.6782 |
| X_2:_ Sample concentration | 0.0566 | 0.0005 | 0.0001 |
| X_3:_ Ethanol | 0.3615 | 0.0119 | 0.0079 |
| X_1_ X_2_ | 0.7219 | 0.9845 | 0.8936 |
| X_1_X_3_ | 0.1722 | 0.4763 | 0.3400 |
| X_2_X_3_ | 0.4081 | 0.0156 | 0.6318 |
| X_1_^2^ | 0.1543 | 0.1224 | 0.6664 |
| X_2_^2^ | 0.9440 | 0.0212 | 0.0773 |
| X_3_^2^ | 0.5319 | 0.0462 | 0.8726 |
| R^2^ | 0.91 | 0.94 | 0.96 |

Data are presented as mean ± SD, (n = 3). Statistical analysis was performed using two-way ANOVA that follows by Tukey’s post hoc test (*p* < 0.05).

**Table S5** Experimental values of DPPH and FRAP under the optimized conditions

|  | | **Ethanol extract** | |
| --- | --- | --- | --- |
|  | | **DPPH (%)** | **FRAP (%)** |
| Optimized conditions | Time (mins) | 60 | 30 |
|  | Sample concentration (µg/mL) | 250 | 50 |
|  | Ethanol (%) | 90 | 50 |
| Response variables | RMSE (%) | 4.77 | 7.21 |

**Table S6** List of hit compounds that passed through the Lipinski rule of five

| **Hits / Control** | **Molecular weight** | **PubChem ID** | **Lipinski** | **GI absorption** | **BBB permeant** | **#H-bond acceptors** | **#H-bond donors** | **PAINS #alerts** | **LogP** | **TPSA (Å²)** |
| --- | --- | --- | --- | --- | --- | --- | --- | --- | --- | --- |
| Hit 1 | 100.07 | 74088 | 0 violation | High | No | 3 | 1 | 0 | -0.21 | 54.37 |
| Hit 2 | 100.07 | 21076 | 0 violation | High | No | 3 | 1 | 0 | -0.12 | 46.53 |
| Hit 3 | 100.12 | 638122 | 0 violation | High | Yes | 2 | 1 | 0 | 0.90 | 37.30 |
| Hit 4 | 100.12 | 6656 | 0 violation | High | Yes | 2 | 1 | 0 | 0.83 | 37.30 |
| Hit 5 | 100.12 | 643915 | 0 violation | High | Yes | 2 | 1 | 0 | 0.84 | 37.30 |
| Hit 6 | 100.12 | 81326 | 0 violation | High | Yes | 2 | 1 | 0 | 0.79 | 37.30 |
| Hit 7 | 106.13 | 18566 | 0 violation | High | Yes | 2 | 0 | 0 | 0.61 | 47.58 |
| Hit 8 | 203.66 | 59857 | 0 violation | High | Yes | 3 | 0 | 0 | 1.59 | 29.54 |
| Hit 9 | 100.16 | 53167 | 0 violation | Low | No | 2 | 1 | 0 | 0.10 | 15.27 |
| Hit 10 | 129.20 | 22212772 | 0 violation | High | No | 2 | 1 | 0 | 0.67 | 23.47 |
| Hit 11 | 214.09 | 545281 | 0 violation | High | Yes | 2 | 2 | 0 | 1.50 | 49.33 |
| Reference 1 | 366.5 | 9543473 | 1 violation | Low | No | 4 | 6 | 0 | 1.02 | 227.10 |
| Reference 2 | 408.25 | 54759160 | 0 violation | High | No | 4 | 2 | 0 | 1.92 | 106.84 |


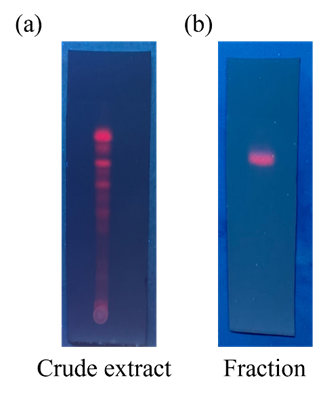


**Fig. S1** TLC profiling of ethanol extract **(a)** Crude ethanol extract and **(b)** Eluted fraction


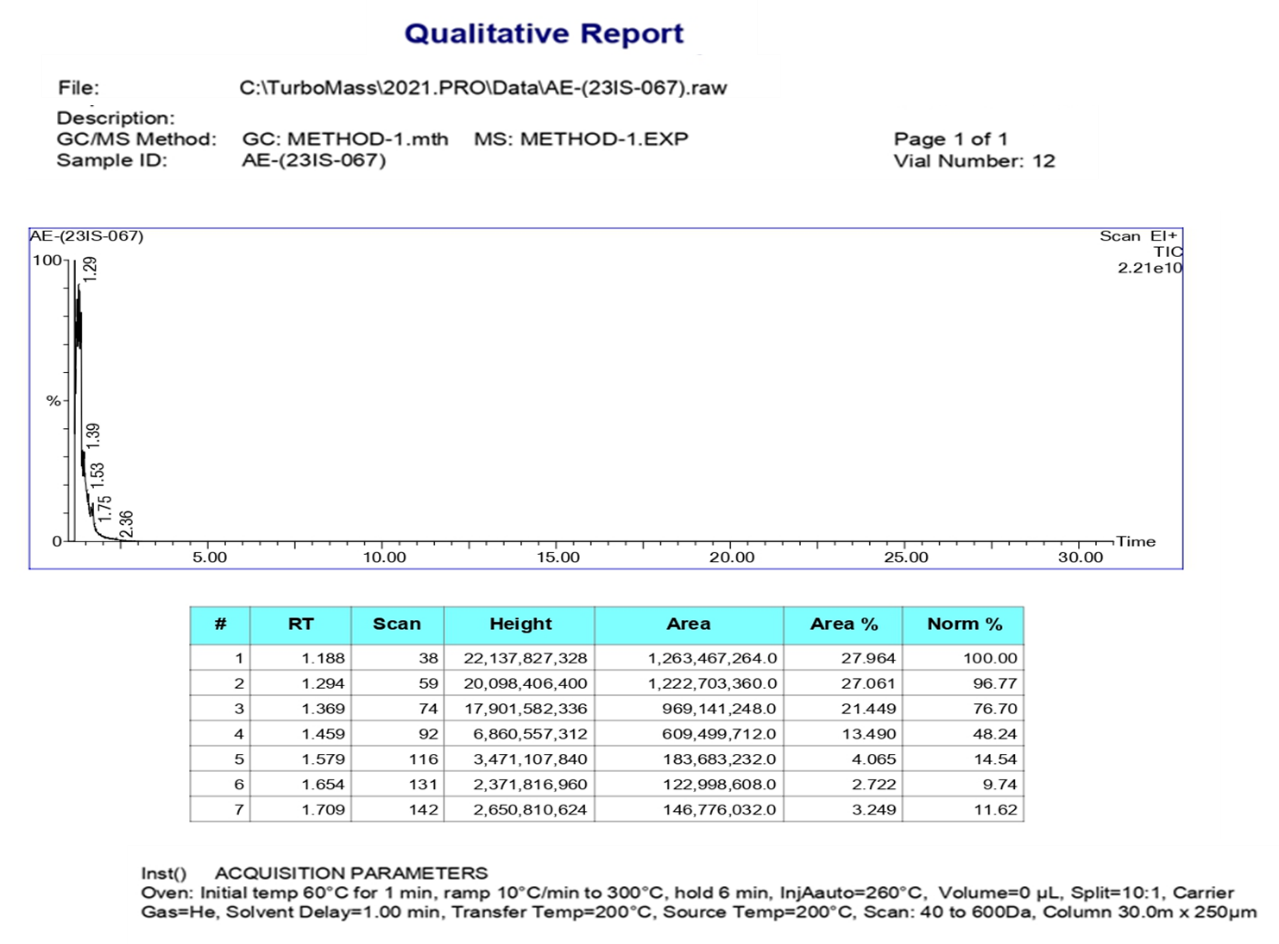


**Fig. S2** GC-MS chromatogram of *A. indica* fraction to identified phytochemical components
